# Organic Matter in Cometary Environments

**DOI:** 10.3390/life11010037

**Published:** 2021-01-08

**Authors:** Adam J. McKay, Nathan X. Roth

**Affiliations:** 1 Department of Physics, American University, Washington, DC 20016, USA; 2 Planetary Systems Laboratory Code 693, Solar System Exploration Division, NASA Goddard Space Flight Center, Greenbelt, MD 20771, USA; 3 Astrochemistry Laboratory Code 691, Solar System Exploration Division, NASA Goddard Space Flight Center, Greenbelt, MD 20771, USA; nathaniel.x.roth@nasa.gov; 4 Universities Space Research Association, Columbia, MD 21046, USA

**Keywords:** comet, organics, volatiles, astrobiology

## Abstract

Comets contain primitive material leftover from the formation of the Solar System, making studies of their composition important for understanding the formation of volatile material in the early Solar System. This includes organic molecules, which, for the purpose of this review, we define as compounds with C–H and/or C–C bonds. In this review, we discuss the history and recent breakthroughs of the study of organic matter in comets, from simple organic molecules and photodissociation fragments to large macromolecular structures. We summarize results both from Earth-based studies as well as spacecraft missions to comets, highlighted by the Rosetta mission, which orbited comet 67P/Churyumov–Gerasimenko for two years, providing unprecedented insights into the nature of comets. We conclude with future prospects for the study of organic matter in comets.

## 1. Introduction

**Figure 1:**
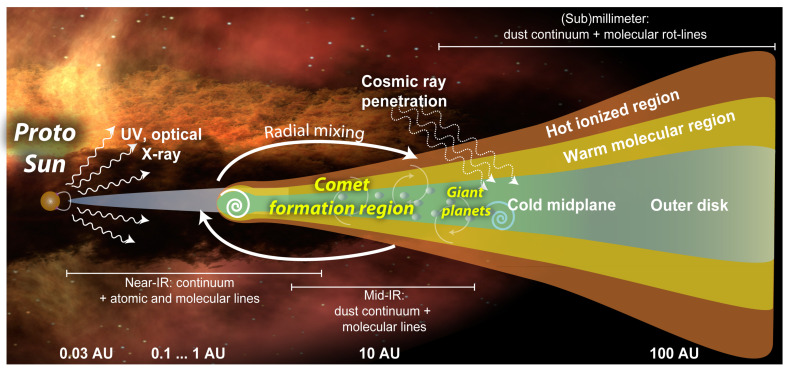
Depiction of a protoplanetary disk and the relevant physical processes. As remote observations of other protoplanetary disks often cannot penetrate deep into the disk, comets serve as our best probes for the physics and chemistry occurring in the cold midplane. Image Credit: Geronimo Villanueva, priv. communication.

Comets are primitive leftovers from the formation of the Solar System. As such, their composition provides clues to physics and chemistry operating during the protoplanetary disk phase (Figure 1), as well as the preceding phases of star formation (e.g., [1,2]). Cometary nuclei consist of a combination of volatile ices and refractory carbonaceous material and silicates [3]. The exact partition (whether comets are “dirty iceballs” or “icy dirtballs”) is still under debate, though results from the recent European Space Agency (ESA) mission Rosetta and other arguments favor an ice-to-dust mass ratio greater than unity (i.e., “icy dirtballs”) [4].

The volatile composition of comets is usually dominated by water (H${}_{2}$O), with carbon dioxide (CO${}_{2}$) and carbon monoxide (CO) also being abundant at the 1–20% level compared to H${}_{2}$O [5,6]. One of the surprising results from the Rosetta mission was the discovery that molecular oxygen (O${}_{2}$) was very abundant in 67P/Churyumov-Gerasimenko (hereafter 67P), with O${}_{2}$/H${}_{2}$O = 110% [7]. However, comets are also rich in simple organic species, such as methanol (CH${}_{3}$OH), ethane (C${}_{2}$H${}_{6}$), methane (CH${}_{4}$), and hydrogen cyanide (HCN). These species have been detected in comets since the 1990s using observations at infrared (IR) and sub-mm wavelengths [5,8]. Simpler radical species such as diatomic carbon (C${}_{2}$) and cyanide (CN) have been detected in comets at optical wavelengths since the 19th century. These species are likely released from the photodissociation of molecules such as acetylene (C${}_{2}$H${}_{2}$) and HCN, though the origin of many of these species is still not completely understood [6,9]. More recently, much more complex organic molecules such as ethanol (C${}_{2}$H${}_{5}$OH), formamide (NH${}_{2}$CHO), glycolaldehyde (CH${}_{2}$OHCHO), and acetaldehyde (CH${}_{3}$CHO) have been detected at sub-mm wavelengths (e.g., [10]). Even glycine, the simplest amino acid, and phosphorous (P, predominantly traced back to PO [11]) were detected by the Rosetta mission [12].

The carbonaceous refractory phase of cometary material also potentially contains organic material, though this phase of cometary material is less understood than the volatile phase. There is observational evidence for polycyclic aromatic hydrocarbons (PAHs) present in cometary material (e.g., [13,14,15,16,17,18,19]). In the past, the presence of PAHs in comets has been highly debated, though the detection of toluene at 67P by Rosetta [20] has provided a firm basis for PAHs being present in comets. The Giotto mission flyby of comet 1P/Halley detected the presence of so-called CHON particles (so named because they were rich in carbon, hydrogen, oxygen, and nitrogen) [21]. These CHON particles have been proposed as a possible source for carbon-bearing radicals like C${}_{2}$ observed at optical wavelengths discussed above (e.g., [22,23]), as well as more complex molecules such as formaldehyde (H${}_{2}$CO) [24].

As the chemical inventory of species detected in comets is rich in organic molecules, comets are of immense interest as possible sources of Earth’s organic material. For the purposes of this review, we adopt the strict chemical definition of organic matter, which encompasses molecules that contain C–H and/or C–C bonds, and focus on species that fit this definition. However, it should be noted that comets are also rich in non-organic species, such as carbon monoxide (CO), carbon dioxide (CO${}_{2}$) and ammonia (NH${}_{3}$), that, despite not being strictly organic, are still important in the formation of prebiotic molecules. In this review, we discuss the history of the study of organic matter in comets, recent advances in our understanding of cometary organic matter, and future prospects for the continued study of organic molecules in cometary nuclei.

## 2. Tools for Studying Cometary Organic Matter

Studies of cometary composition are often limited to remote sensing of the gas-phase coma (transient gravitationally unbound atmosphere) that surrounds the nucleus of the comet, owing to sublimation of the primary ices H${}_{2}$O, CO${}_{2}$, and CO. These methods employ spectroscopy at a variety of wavelengths to observe electronic, vibrational, rotational, and rovibrational transitions of molecules of interest in the gas phase. More recently, spacecraft missions have enabled studies of the solid phase of cometary material, most notably through sample return of refractory material (dust) by the Stardust mission and the lander Philae (associated with the Rosetta mission), which was able to perform in situ analysis on the comet’s surface. In situ missions also sample the coma gas through mass spectroscopy. We discuss these methods below.

### 2.1. Remote Sensing

#### 2.1.1. Optical

**Figure 2:**
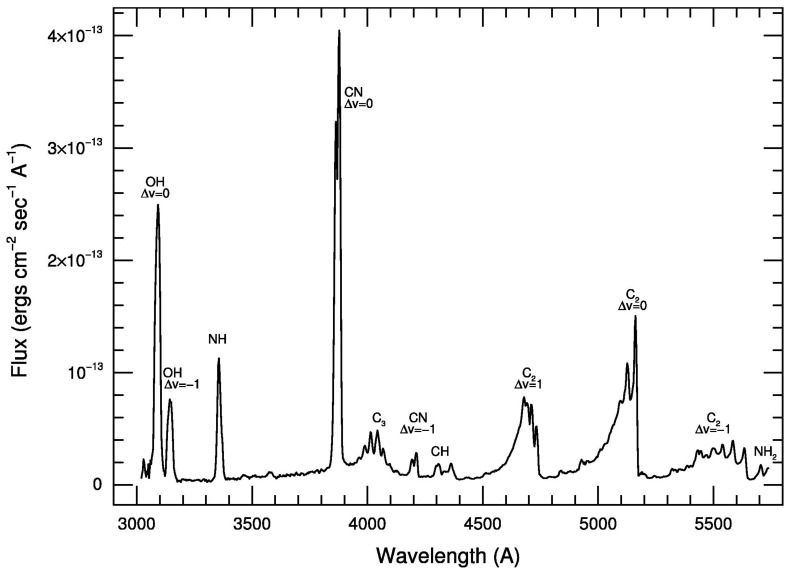
Optical spectrum of comet 122P/De Vico showing typical emissions observed in cometary spectra. Many of these species are likely released via the photodestruction of simple organic matter or carbon-rich dust grains. Figure from [29].

Optical observations of comets (which we define as covering wavelengths from 300–1000 nm) have the longest history, with the first spectra of comets being obtained in the 19th century [25,26,27]. Comets show a rich emission line spectrum at optical wavelengths, dominated by emissions from CN and C${}_{2}$ (see Figure 2). Many of these molecules are carbon-bearing and are likely fragments of more complex organic matter present in cometary ices and dust. Narrowband filter sets have also been developed so that imaging techniques can be used to isolate molecules of interest [28].

Over 200 comets have been characterized at optical wavelengths (e.g., [29,30,31,32]). These studies have revealed several taxonomic groups, including comets that are depleted in carbon-chain species, and provided the first glimpse into variations in composition among comets (see Section 3.9.2). However, due to the fragment nature of the observed species and our lack of understanding of the coma processes responsible for their release [9,33], interpretation of these taxonomic groupings in terms of the more complex organic matter that they likely trace is difficult.

Optical spectroscopy at high spectral resolution has also proven to be a powerful method for determining isotopic ratios for C and N in cometary material. The first isotopic measurements in C and N came from the CN molecule [34,35], with some carbon measurements also coming from observations of C${}_{2}$ [36]. More recently, NH${}_{2}$ has been utilized to measure isotopic ratios in N [37,38,39]. These measurements revealed Earth-like ratios in C, but ratios in N deviate from the terrestrial value by a factor of two and solar value by a factor of three. The reason for this discrepancy is not fully understood (see Section 3.9.1).

#### 2.1.2. Near Infrared (NIR)

**Figure 3:**
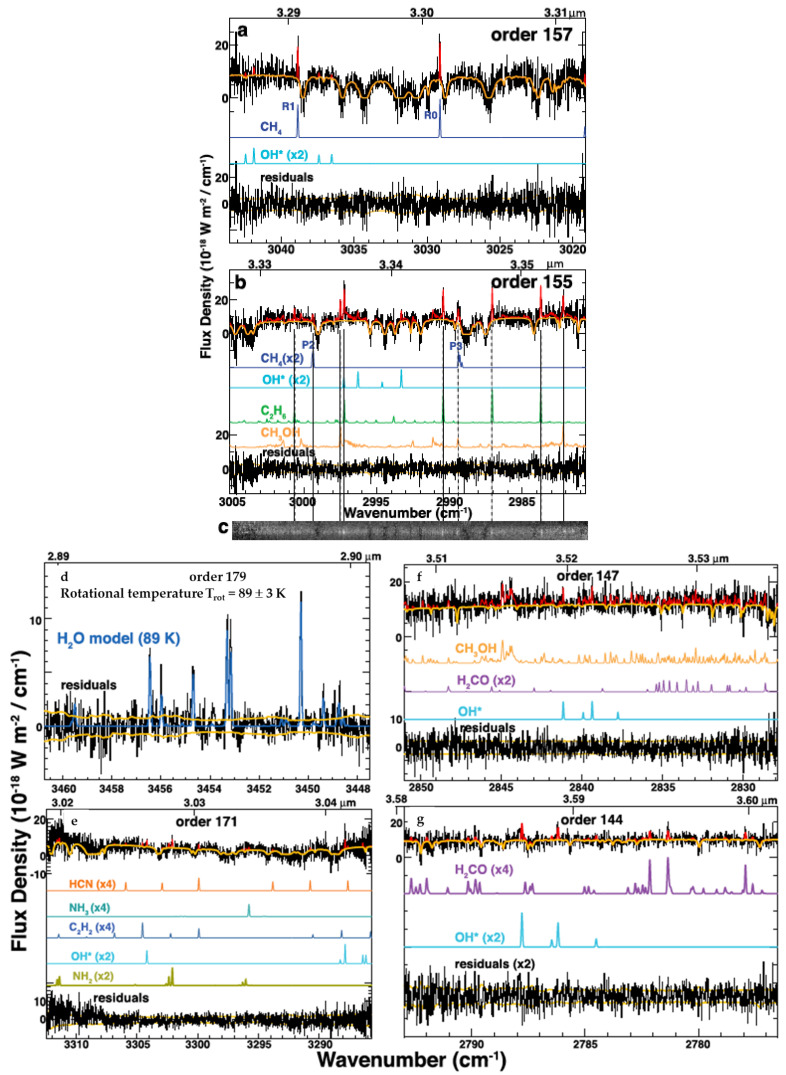
IR spectra of comet 45P/Honda-Mrkos-Pajdušáková obtained with iSHELL on the NASA Infrared Telescope Facility (IRTF) showing spectral regions containing typical emissions observed in cometary spectra. These include CH${}_{4}$ (**a** and **b**), C${}_{2}$H${}_{6}$ (**b**), CH${}_{3}$OH (**b** and **c**), H${}_{2}$O (**d**), H${}_{2}$CO (**f** and **g**), HCN, C${}_{2}$H${}_{2}$, and NH${}_{3}$ (**e**). In particular, high spectral resolution IR spectroscopy is the only method to remotely observe symmetric hydrocarbons like C${}_{2}$H${}_{6}$ and CH${}_{4}$ in comets due to their lack of pure rotational transitions. The observed spectra are in black, with fluorescence models overplotted in red and fits for individual species offset below the observed spectrum. The “*” after OH in the model labels indicates these emissions are prompt emission, while all other emissions are fluorescence. Figure from [52].

NIR observations of comets were pioneered in the 1980s, resulting in the first direct detection of H${}_{2}$O from a comet [40]. The development of ground-based high spectral resolution instruments in the 1990s resulted in the first observations of a wider volatile composition of comets at IR wavelengths (e.g., [41,42]), with individual organic molecules such as CH${}_{4}$, C${}_{2}$H${}_{6}$, and acetylene (C${}_{2}$H${}_{2}$) identified for the first time (see Figure 3). NIR observations at high spectral resolution are the only way to directly study symmetric hydrocarbons such as C${}_{2}$H${}_{6}$, C${}_{2}$H${}_{2}$, and CH${}_{4}$ in comets with remote facilities, as these species lack a permanent dipole moment and pure rotational transitions, precluding their measure at millimeter/sub-millimeter wavelengths (see Section 2.1.3). Low spectral resolution IR observations are also diagnostic of organic matter, but to date have mostly been applied to observations of cometary surfaces visited by spacecrafts (see Section 2.2).

#### 2.1.3. Mm/Sub-mm

**Figure 4:**
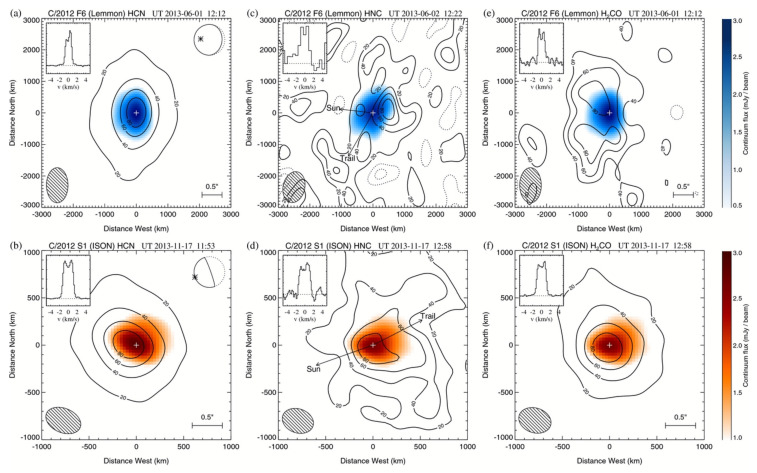
ALMA maps of the spatial distribution of HCN (**a**,**b**), HNC (**c**,**d**), and H${}_{2}$CO (**e**,**f**) in two comets: C/2012 F6 (Lemmon) (**a**,**c**,**e**) and C/2012 S1 (ISON) (**b**,**d**,**f**). The contours represent the line emission of the respective gas species, while the color scale shows the continuum emission from the dust coma. Figure from [48].

Observations at millimeter (mm) and submillimeter (sub-mm) wavelengths have a similar history to the NIR, with observations dating as far back as the 1970s (e.g., [43,44]). Advances in instrumentation in the 1990s first enabled the regular detection of a suite of molecules in comets at mm/sub-mm wavelengths. These observations have the advantage of being sensitive to more complex species than are observed at optical/NIR wavelengths, such as formamide and ethanol, through their rotational transitions. Sub-mm observations also have higher spectral resolution than optical/IR observations, enabling velocity-resolved studies of coma species via the analysis of their spectral line profiles. These observations measure molecular outflow velocities, and can reveal potential coma outgassing asymmetries. However, this often comes at the expense of spatial resolution on the sky. Early cometary observations were dominated by single-dish telescopes (e.g., [45,46,47]) with an angular resolution on the scale of several to tens of arcseconds (corresponding to thousands of kilometers projected distance at the comet). The development of interferometers, including, but not limited to, the Atacama Large Millimeter/Submillimeter array (ALMA), with sub-arcsecond spatial resolution has revolutionized the study of organic molecules in comets, providing spatially resolved maps of the distributions of species such as HCN, H${}_{2}$CO, and CH${}_{3}$OH (see Figure 4) (e.g., [48,49,50]).

### 2.2. Spacecraft Missions

Spacecraft missions to comets have proven invaluable to the study of comets in general and specifically organic matter in comets, providing observations of the nucleus and compositional information only attainable in situ. Below we highlight a few missions with a particularly large impact on our knowledge of organic matter in comets.

#### 2.2.1. The Halley Armada

The first comet to be studied via spacecraft flybys of the nucleus was 1P/Halley in 1986. A suite of spacecrafts flew by the comet, including ESA’s Giotto mission and the Soviet probe Vega 2. Giotto carried a mass spectrometer that measured the composition of the coma in situ for the first time [51]. It also obtained IR spectra showing the presence of H${}_{2}$O, CO, CO${}_{2}$, and organic matter [13]. Vega 2 provided optical and IR spectra of the inner coma, providing the first evidence for the presence of PAHs in comets (e.g., [18]).

#### 2.2.2. Stardust

NASA’s Stardust mission is to date the only mission to return cometary material for analysis in Earth-based laboratories [53]. The spacecraft performed a flyby of comet 81P/Wild 2 on 2 January 2004, collecting dust particles from the coma in aerogel. These samples were then successfully returned to Earth two years later on 15 January 2006. With the ability to analyze the collected dust particles in state-of-the-art Earth-based laboratories rather than the necessarily limited instruments on a spacecraft, a wealth of studies have been performed, ranging from isotopic composition to the presence of organic material (e.g., [16,53,54]), including the amino acid glycine [55]. Related to these particles is the study of interplanetary dust particles (IDPs), which are collected from the upper layers of the Earth’s atmosphere (e.g., [56,57]). These particles may very well have a cometary origin, but determining a definitive link to a specific comet is difficult, though it has been attempted (e.g., [58]).

#### 2.2.3. Rosetta

**Figure 5:**
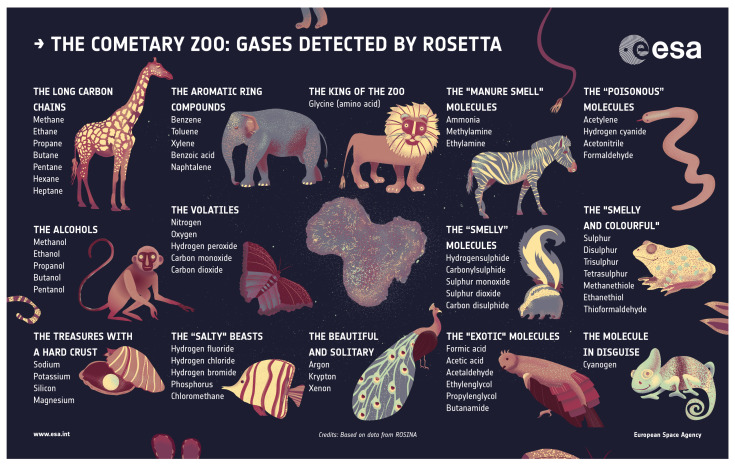
A graphic representation of all gases detected by the Rosetta mission, depicted here as the “Cometary Zoo”. Many of these species are organic molecules and were detected for the first time in a cometary coma by Rosetta. Image Credit: ESA.

**Figure 6:**
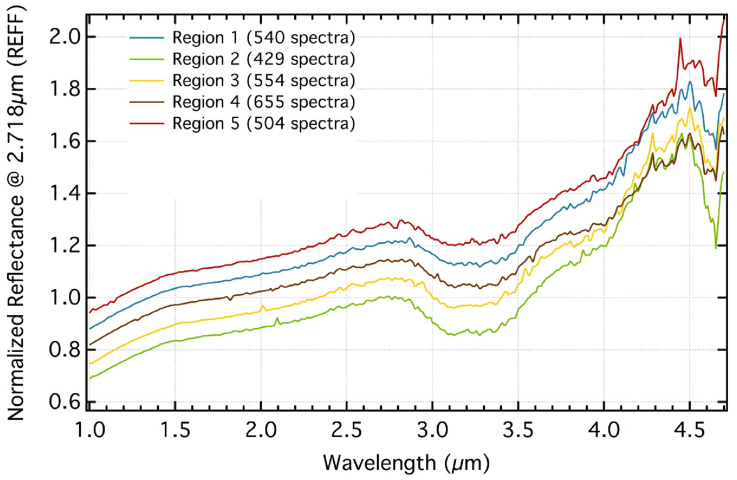
Spectra of several regions on the surface of 67P showing strong absorption at 3.0–3.5 μm, which is attributable in part to the presence of organic matter. Figure from [63].

The Rosetta mission provided huge leaps forward in our understanding of comets and their molecular composition (see Figure 5). Impactful instruments of the many on board include the Rosetta Orbiter Spectrometer for Ion and Neutral Analysis (ROSINA) mass spectrometer and the Visible and Infrared Thermal Imaging Spectrometer (VIRTIS) IR spectrometer, as well as the Cometary Sampling and Composition (COSAC) and Ptolemy mass spectrometers on the Philae lander. ROSINA was able to perform an in situ analysis of the coma gas around the target comet, 67P/Churyumov-Gerasimenko. These observations provided extensive measurements of species previously known in comets, but also discovered many new species never before detected in comets. These include complex organic molecules that are beyond the grasp of current Earth-based observation techniques, such as glycine [12], due to their low abundance and complex spectra. COSAC and Ptolemy on the Philae lander were able to perform a similar in situ analysis of the surface material. Although the Philae lander only operated for a few days due to power constraints created by a non-optimal landing configuration, COSAC and Ptolemy still provided the first measurements directly from the surface of a cometary nucleus (see [59] for a complete review of Philae results). The VIRTIS instrument provided NIR spectroscopy of the surface of 67P revealing the presence of water and CO${}_{2}$ ice [60,61,62], as well as an organic absorption feature around 3.2 microns ([63,64], see Figure 6).

## 3. Results for Specific Species

Table 1 lists the organic molecules detected via the Earth-based remote sensing of cometary comae with information pertaining to the number of comets in which this species has been detected and typical abundances. For a list of additional organic species detected by Rosetta and Philae, see Table 4 of Altwegg et al. 2019 [65]. Below, we summarize recent results pertaining to specific species or types of organic matter detected in comets. This includes parent (or primary) species that are inherently present in the nucleus, as well as daughter (or secondary) species that are produced by photolysis in the coma. More details regarding the volatile composition of comets can be found in recent reviews/survey studies (e.g., [5,6,8,65,66,67,68]).

### 3.1. HCN and HNC

Hydrogen cyanide (HCN) is a key precursor in the synthesis of amino acids and is likely integral to the presence of life on Earth [69]. It was first securely detected in comet 1P/Halley [70,71] and is routinely sampled in comets with state-of-the-art instruments, through its intrinsically strong pure rotational and ro-vibrational transitions in the mm/sub-mm and in the near-infrared, respectively. HCN is one of the main reservoirs of volatile nitrogen in comets (as opposed to the total nitrogen, which may be locked up in more refractory material such as ammonium salts [72,73]) and a likely parent of the fragment species CN (see Section 3.9.1) [23,74,75]. Long-slit near-IR observations (e.g., [8]) and spatially resolved interferometric studies [48] have indicated that the distribution of HCN is consistent with direct sublimation from the nucleus, (i.e., a parent species, see Figure 4). Interestingly, HCN abundances as measured in the near-IR for a given comet can be two to three times higher than those measured at mm/sub-mm wavelengths [67], even when comparing to ALMA measurements with a beam size comparable to that of near-IR instruments. The reason for this discrepancy is not understood. Depending on the wavelength of the study, average HCN abundances compared to H${}_{2}$O in comets range from 0.1–0.2% [67,75].

Hydrogen isocyanide (HNC), an isomer of HCN, was first detected in comet C/1996 B2 (Hyakutake) [76], with an HNC/HCN abundance ratio in agreement with interstellar values. However, the strong heliocentric dependence of the HNC/HCN ratio in C/1995 O1 (Hale–Bopp) [77] indicated that cometary HNC is more consistent with production from coma sources. Various formation mechanisms, from the isomerization of HCN to thermal degradation of polymers [78,79] have been proposed. A review of 11 comets confirmed a strong heliocentric dependence in the HNC/HCN ratio that was incompatible with direct release from the nucleus [80]. More recent work with ALMA in comet C/2012 S1 (ISON) revealed HNC production following the release of a clump of ice- or organic-rich material from the nucleus [49]. Owing to the absence of the clump in simultaneously observed images of H${}_{2}$CO or CH${}_{3}$OH, it was concluded that the clump was more likely rich in polymeric or macromolecular organic matter, making the HNC parent more consistent with a refractory component (see Section 3.8) than an ice one.

### 3.2. H${}_{2}$CO

Formaldehyde (H${}_{2}$CO) is a chemical precursor to sugars, and similar to HCN, can be measured with both mm/sub-mm and NIR studies. It was first unambiguously detected at mm wavelengths in comets C/1990 V (Austin) and C/1990 XX (Levy) [81,82], although tentative detections were claimed during flyby and ground-based observations of 1P/Halley [13,83,84,85]. Measurements of H${}_{2}$CO${}^{+}$ by the neutral mass spectrometer on board the Giotto spacecraft provided the first indication of H${}_{2}$CO production by an unknown parent in the coma [86]. More recent studies of H${}_{2}$CO at mm wavelengths in comets indicated production from unknown parent source(s) with a scale length of 7000 km at 1 AU [24,45,87], and sub-mm ALMA observations of the inner coma indicate a parent scale length of 1000–5000 km [48,49]. On the other hand, NIR observations, which are most sensitive to the near-nucleus region, have indicated that H${}_{2}$CO can also be released directly from the nucleus [75,88], suggesting that both nucleus and coma sources can contribute to H${}_{2}$CO production in comets. Regardless of the nature of its release, H${}_{2}$CO has abundances ranging from 0.04–1.3% with respect to H${}_{2}$O in comets measured to date [67,75].

Collectively, these results have shown that H${}_{2}$CO is one of a number of molecules which is inconsistent with direct release from the nucleus alone and is likely produced by unidentified coma sources (“distributed” sources; [89]). The visible, infrared and thermal imaging spectrometer (VIRTIS) instrument onboard Rosetta detected organic macromolecular material that is likely the source for some of these unidentified parents [90]. Degradation of the H${}_{2}$CO polymer, polyoxymethylene (POM), was proposed to explain the heliocentric dependence of H${}_{2}$CO production in comet C/1995 O1 (Hale–Bopp) [91] and may be the unidentified H${}_{2}$CO parent, though this has yet to be confirmed (see Section 3.8). Analysis of the data obtained by the ROSINA instrument onboard Rosetta concluded that the presence of POM is unlikely [20]. Further studies of cometary H${}_{2}$CO, particularly spatially resolved interferometry with facilities such as ALMA combined with new spacecraft missions (particularly volatile sample return), will be necessary to help identify its parent source.

### 3.3. CH${}_{3}$OH

**Figure 7:**
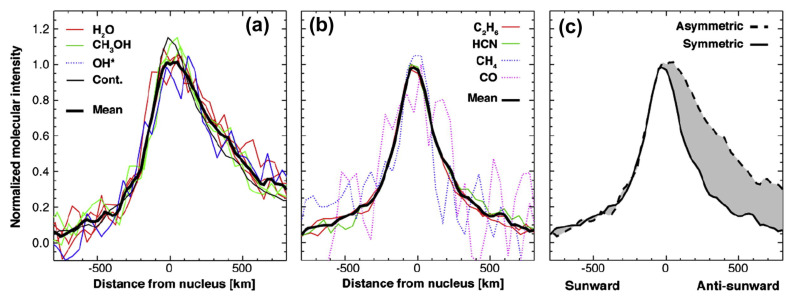
Spatial profiles for various species observed in comet C/2007 W1 (Boattini) at IR wavelengths. Panel (**a**) shows H${}_{2}$O, CH${}_{3}$OH, OH (a tracer for H${}_{2}$O), and the dust continuum, while panel (**b**) shows C${}_{2}$H${}_{6}$, HCN, CH${}_{4}$, and CO. The “*” after the OH label indicates the emissions used to measure the spatial distribution are prompt emission, while all other emissions are fluorescence. Panel (**c**) shows the average profile of the species in each panel, and the grey shading highlights the difference between the two profiles. The species in panel (**a**) are spatially extended in the antisunward direction (positive x values), while the species in panel (**b**) are symmetric. This indicates different modes of release for the different volatiles. Figure from [100].

Methanol (CH${}_{3}$OH) is the simplest alcohol and is the starting point for the formation of more complex organic molecules in the interstellar medium (ISM) [92,93,94]. It was first detected in comets at mm wavelengths [95], and has since been measured in many comets at mm/sub-mm and near-infrared wavelengths [67,75]. The heliocentric dependence of CH${}_{3}$OH production, combined with long-slit near-infrared studies and spatially resolved ALMA observations, has shown that it is primarily associated with direct nucleus release [46,50,75]. However, ground-based measurements of hyperactive comet 103P/Hartley 2 indicated increased anti-sunward CH${}_{3}$OH and H${}_{2}$O production that was associated with sublimation from icy coma grains [96,97,98,99]. This phenomenon was also observed in comet C/2007 W1 (Boattini) (see Figure 7, panel a) [100]. CH${}_{3}$OH is one of the more abundant species in comets, ranging from 0.4–6.2% relative to H${}_{2}$O [67,75].

In addition to its likely origin as a parent molecule in comets, CH${}_{3}$OH has proven invaluable for probing coma temperatures and thermal physics in comets. Multiple CH${}_{3}$OH lines are routinely used to measure coma rotational and/or kinetic temperatures (e.g., [95]). Coupled with spatially resolved sub-mm observations, detailed CH${}_{3}$OH maps can show variations in rotational and kinetic temperature with nucleocentric distance, revealing the thermal physics of the inner coma [50].

### 3.4. C${}_{2}$H${}_{6}$

C${}_{2}$H${}_{6}$ was first detected in a cometary coma in C/1996 B2 (Hyakutake) [101], and is responsible for one of the brightest emission bands observed in comets at NIR wavelengths. It is also a relatively abundant trace species present in comets, with an average abundance of 0.55% compared to H${}_{2}$O, though in the sample of comets studied to date, there is a factor of two difference between the average C${}_{2}$H${}_{6}$ abundance in Oort cloud comets (OCCs, comets whose dynamical reservoir is the Oort cloud) compared to the average value for Jupiter family comets (JFCs, comets whose orbits are dynamically linked to Jupiter and are thought to have been perturbed inward from the scattered disk) [8]. As a symmetric molecule, C${}_{2}$H${}_{6}$ does not have a permanent dipole moment and therefore is not observable at sub-mm wavelengths. C${}_{2}$H${}_{6}$ in comets was likely formed from the addition of hydrogen atoms to simpler hydrocarbons such as C${}_{2}$H${}_{2}$ via grain-surface reactions [101,102,103], but C${}_{2}$H${}_{6}$ can also be formed from the irradiation of CH${}_{4}$ ices [101,104,105]. Irradiation of C${}_{2}$H${}_{6}$ can in turn result in the formation of more complex hydrocarbons [106].

Because of the intrinsic brightness of C${}_{2}$H${}_{6}$ lines, studies of the spatial distribution of C${}_{2}$H${}_{6}$ have been utilized to determine how C${}_{2}$H${}_{6}$ is released into the coma. For example, spatial profiles in comet C/2007 W1 (Boattini) showed a similarity with HCN, yet were different than those observed for H${}_{2}$O and CH${}_{3}$OH, which was interpreted as evidence for these molecules being stored in different ice phases in the nucleus (see Figure 7) [100]. A similar phenomenon was observed for 103P/Hartley 2, prompting the suggestion of an ice phase dominated by polar molecules such as H${}_{2}$O and CH${}_{3}$OH and a different ice phase featuring apolar molecules such as CO${}_{2}$ and C${}_{2}$H${}_{6}$ [96,97,99]. Observations of comet C/2013 V5 (Oukaimeden) with Keck NIRSPEC showed a time variable C${}_{2}$H${}_{6}$/H${}_{2}$O ratio on the order of days, further illustrating that C${}_{2}$H${}_{6}$ and H${}_{2}$O ices are not co-located in the nucleus [107].

### 3.5. CH${}_{4}$

**Figure 8:**
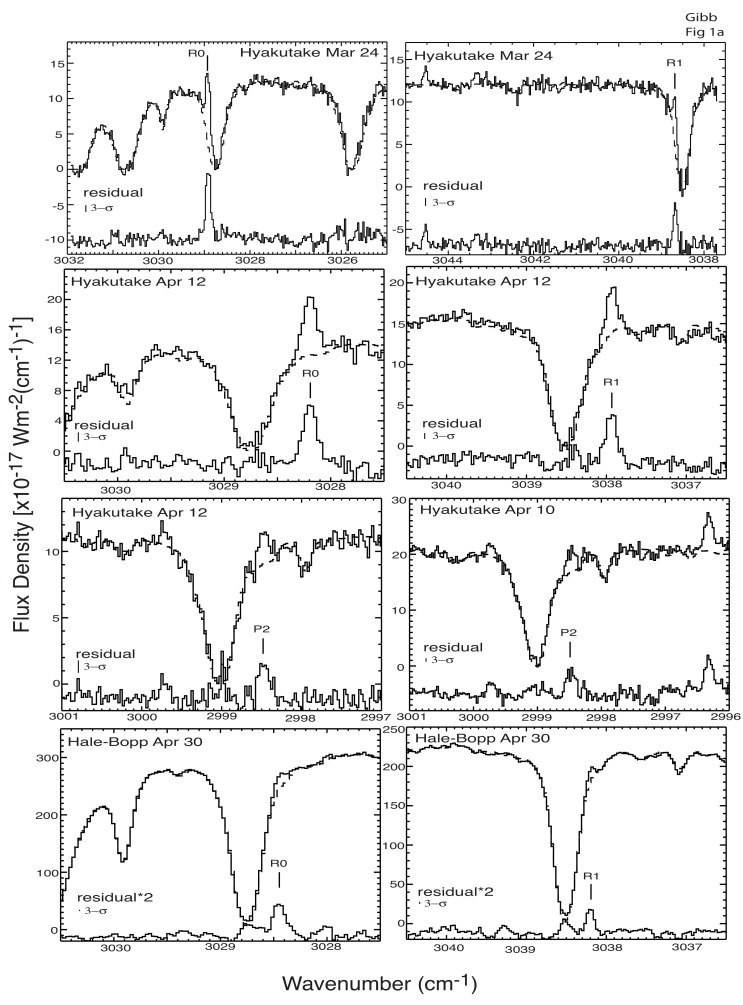
IR spectra showing CH${}_{4}$ emissions in comets C/1996 B2 (Hyakutake) (top six panels) and C/1995 O1 (Hale–Bopp) (bottom two panels). The measured spectra (complete with continuum) are shown above, whereas the continuum removed spectra are shown below in each panel, labeled as residual. For Hale-Bopp, the continuum removed spectra are multiplied by a factor of two, indicated by “residual*2”. The strong absorptions are due to telluric CH${}_{4}$, illustrating the need for cometary emissions to be Doppler-shifted away from the center of these features by sufficient geocentric velocity of the comet. Figure from [128].

Like C${}_{2}$H${}_{6}$, CH${}_{4}$ is a symmetric hydrocarbon and therefore is only observable through its IR rovibrational transitions. However, the CH${}_{4}$ lines observable are intrinsically weaker than C${}_{2}$H${}_{6}$ emissions, making CH${}_{4}$ harder to detect in cometary comae. Moreover, strong CH${}_{4}$ absorptions from the Earth’s atmosphere (telluric absorptions) make it only possible to observe CH${}_{4}$ from the ground for certain observing geometries, requiring a sufficient geocentric velocity to Doppler-shift cometary CH${}_{4}$ emissions away from the corresponding telluric absorptions (see Figure 8). The requirement to achieve this geometry makes observations of CH${}_{4}$ even more limited in comets, especially for JFCs, which are generally fainter and have lower production rates than OCCs and thus must be observed near closest approach to Earth. This necessarily coincides with a low geocentric velocity, precluding the measurement of CH${}_{4}$ and resulting in relatively few measurements of CH${}_{4}$ in JFCs and thus a significant gap in our understanding of its abundance in this dynamical class. Space-based observations do not suffer from this limitation, though to date, space-based platforms have generally not been able to observe CH${}_{4}$ in comets, and the vast majority of observations of cometary CH${}_{4}$ have come from ground-based facilities [8]. An exception is the Rosetta mission, where CH${}_{4}$ was detected using IR observations with the VIRTIS instrument [108] and mass spectrometry with ROSINA [109].

The average CH${}_{4}$ abundance in comets with respect to H${}_{2}$O is 0.78%; though similar to C${}_{2}$H${}_{6}$, there is a strong difference in the mean values between OCC’s (0.88%) and JFC’s (0.31%) [8]. However, as noted above, the statistics for CH${}_{4}$ in JFCs are far from robust. Recent years have provided several rare and favorable opportunities to observe CH${}_{4}$ in JFCs [52,110,111,112,113,114], providing a much needed boost towards establishing a statistically significant sample of CH${}_{4}$ measurements in JFCs. However, it is still much smaller than the OCC sample, making comparisons between the two classes more difficult. Therefore, further studies of CH${}_{4}$ in JFCs are required for definitive comparisons between these two major dynamical families. CH${}_{4}$ is likely formed from hydrogen addition reactions on atomic carbon on dust grain surfaces [115], and, as mentioned above, the irradiation of CH${}_{4}$ ices can lead to the formation of C${}_{2}$H${}_{6}$ and more complex hydrocarbons.

### 3.6. C${}_{2}$H${}_{2}$

C${}_{2}$H${}_{2}$ is another symmetric hydrocarbon observed in cometary comae that has only been detected remotely at IR wavelengths, though it has been detected by ROSINA via mass spectrometry [109]. While its emissions do not suffer from corresponding telluric extinction like CH${}_{4}$ does, lines of cometary C${}_{2}$H${}_{2}$ are generally weak, and it also has a lower mean abundance than either C${}_{2}$H${}_{6}$ or CH${}_{4}$ (average C${}_{2}$H${}_{2}$/H${}_{2}$O = 0.13%, see Table 1), making it more challenging to detect. Similar to C${}_{2}$H${}_{6}$ and CH${}_{4}$, there seems to be a factor of two difference in average C${}_{2}$H${}_{2}$ abundances between JFCs and OCCs, with JFCs having lower abundances. C${}_{2}$H${}_{2}$ can be formed in the ISM through reactions between atomic carbon and molecular hydrogen [116], and like C${}_{2}$H${}_{6}$, can serve as the starting point for the formation of complex hydrocarbons such as benzene [117,118]. C${}_{2}$H${}_{2}$ is the most likely volatile parent molecule for the C${}_{2}$ radical, but often is not the sole source of C${}_{2}$ (see Section 3.9.2).

### 3.7. Other Organic Molecules

In addition to the commonly detected molecules detailed above, other complex organic molecules have been detected in comets. For ground-based observations, this has been limited to the brightest comets, such as C/1995 O1 (Hale–Bopp) and C/2014 Q2 (Lovejoy). These larger molecules are accessed via their rotational transitions at mm/sub-mm wavelengths. These include CHO-bearing molecules such as formic acid (HCOOH), methyl formate (HCOOCH${}_{3}$), ethylene glycol ((CH${}_{2}$OH)${}_{2}$), acetaldehyde (CH${}_{3}$CHO), ethanol (C${}_{2}$H${}_{5}$OH), and the simplest monosaccharide sugar, glycolaldehyde (CH${}_{2}$OHCHO). It also includes nitrogen-bearing species such as isocyanic acid (HNCO), cyanoacteylene (HC${}_{3}$N), acetonitrile (CH${}_{3}$CN), and formamide (NH${}_{2}$CHO), as well as the sulfur-bearing organic molecule thioformaldeyde (H${}_{2}$CS) [10,87,119,120]. Owing to them being measured in so few comets, their abundances across the population are very poorly constrained, yet their detection illustrates the record of complex chemistry stored in cometary nuclei. Furthermore, the in situ results from both the mass spectrometer ROSINA aboard Rosetta, as well as COSAC and Ptolemy on the Philae lander, revealed a suite of complex organic molecules in 67P never before detected in comets. Philae was able to detect a number of complex organic molecules only observed in bright comets remotely, such as acetonitrile and ethylene glycol, as well as organic molecules not previously detected in comets, such as acetone ((CH${}_{3}$)${}_{2}$CO) [121]. Many of these molecules were also detected in the coma by ROSINA [20].

The amino acid glycine was detected in samples returned from 81P by the Stardust mission [55]. Glycine was also detected around 67P by Rosetta [12], and further analysis showed that glycine had a distributed source, consistent with release from dust grains in the coma [122].

### 3.8. Complex Hydrocarbons, PAHs, and CHON Particles

The first evidence for complex macromolecular organic matter dates back to the Halley flybys, with spacecraft determining the presence of complex organic matter in the coma (often referred to as CHON particles) [21]. The Giotto mission also found evidence for a distributed source of H${}_{2}$CO (i.e., H${}_{2}$CO not released directly from the nucleus). This was initially viewed as evidence for the presence of POM [86] (Section 3.2). Results from the Ptolemy instrument on the Philae lander on comet 67P from the Rosetta mission showed evidence for POM [123], however measurements from the spacecraft in the coma from ROSINA could not confirm this finding [20]. Evidence for the presence in the dust grains of large macromolecular organic matter similar to that in meteorites was found by Rosetta [124].

The presence of PAHs in comets has been a subject of some controversy. Optical and IR spectra obtained by the Giotto and Vega 2 flybys showed evidence for emissions that could be assigned to PAHs (e.g., [13,18]). Spitzer observations revealed candidate PAH emissions from comet 9P/Tempel 1 [15] after the Deep Impact experiment, which launched a copper impactor into the surface of the comet in order to excavate material from the subsurface to be studied [125]. More recent ground-based studies have found unidentified emission features in the IR spectra of comet 21P/Giacobini-Zinner, which were interpreted as possible emissions from PAHs [19]. IR spectroscopy of the surface of 67P by VIRTIS on Rosetta revealed spectral signatures consistent with PAHs, though the more readily identified features are aliphatic [64]. The PAH toluene was definitively detected by ROSINA and tentatively detected by Ptolemy on the Philae lander at 67P [20], providing the most firm evidence to date of PAHs in comets.

**Figure 9:**
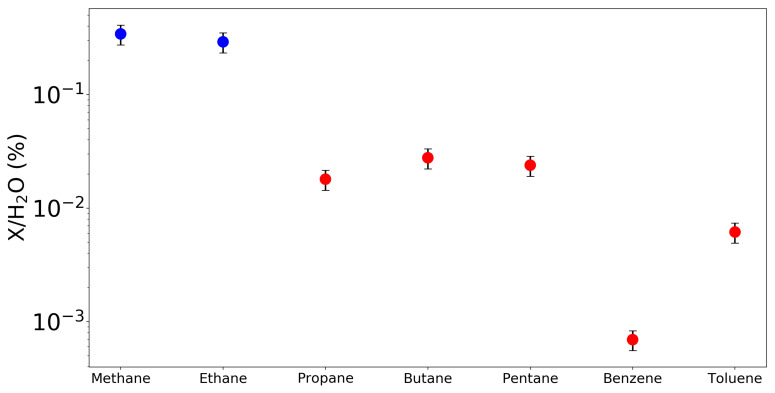
Plot showing the abundances compared to H${}_{2}$O of various hydrocarbons detected by ROSINA in the coma of 67P at a heliocentric distance of 1.52 AU in May 2015. While butane and pentane were not detected in May 2015, they were detected in May 2016, when all hydrocarbons abundances relative to water were significantly higher than in May 2015. To account for this, the values plotted are their May 2016 measurements scaled by their mixing ratios compared to methane. Blue points represent species previously detected in comets, while red points denote species not previously detected, demonstrating the dramatic increase in known hydrocarbons provided by Rosetta. Figure created using data from [127] and based on Figure 8 from [65].

The Stardust and Rosetta missions provided further evidence for complex refractory organic matter in comets. Particles returned by the Stardust mission contained complex organic matter [16,126]. Evidence for both aliphatic and aromatic hydrocarbons was provided both by mass spectrometry in the coma [127], as well as IR spectroscopy of the surface [64]. These hydrocarbons included species as complex as toluene and heptane (see Figure 9) [127].

### 3.9. Organic Tracers

While optical observations are not sensitive to parent organic molecules that are observed at IR and sub-mm wavelengths, they provide abundances of simpler radical species (CN, C${}_{2}$, CH, and C${}_{3}$) that are produced via the photochemistry of organic matter (see Figure 2). As the sample size of optical observations is much larger than IR/sub-mm observations (>200 comets in the optical vs. ∼50 in the IR/sub-mm), they represent the most extensive database for the organic composition of comets, even if linking their abundances to parent organic molecules is not straightforward. In this section, we describe the current knowledge of these organic tracers and their links to parent organic molecules.

#### 3.9.1. CN

Emission from the CN radical is a very bright, nearly ubiquitous feature in cometary spectra at optical wavelengths and has been observed since spectra of comets were first obtained in the 1800’s [25,26,27,129]. CN has also been observed in the NIR, though interpretation of these emissions in terms of abundances has been limited [75]. As a radical species, it is not inherently present in the nucleus, but is released into the coma via photodissociation of a more complex organic. The leading candidate for this molecule is HCN, though other molecules such as C${}_{2}$N${}_{2}$ and HC${}_{3}$N have been suggested, even though currently, there are little observational data constraining the abundances of these species in cometary comae [130]. While HCN can explain the abundance of CN in many comets (e.g., [23]), there are examples of comets where the CN abundance exceeds that of HCN, and therefore HCN cannot account for all the observed CN (e.g., [74,75]). In these cases, the sublimation of carbon-rich dust grains or CHON particles is often invoked. While CN is easily detected in comets, the lack of knowledge of its origin hinders interpretation of its abundance in terms of nucleus composition.

**Figure 10:**
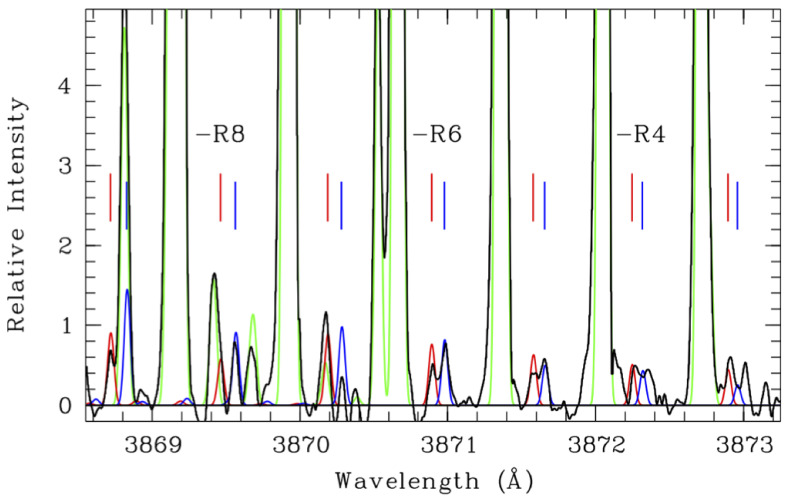
High spectral resolution optical spectrum showing individual CN lines, both of the main isotopologue as well as ${}^{13}$CN and C${}^{15}$N. The data are shown in black, with model fits to the observed emission for the main isotopologue (green), ${}^{13}$CN (blue), and C${}^{15}$N (red) overplotted. The R8, R6, and R4 labels indicate the rotational designation for the observed lines. This demonstrates the neccessity and power of high spectral resolution for isotopic studies of CN at optical wavelengths. Figure from [134].

Due to its intrinsic brightness, CN optical emission has been a favored target for studies of the carbon and nitrogen isotopic ratios in comets, and is the most efficient way to determine isotopic ratios in cometary organic matter via remote sensing. High spectral resolution (λ/Δλ> 30,000) is required to separate ${}^{13}$CN and C${}^{15}$N emission lines from the main isotopologues (see Figure 10). The ${}^{12}$C/${}^{13}$C ratio in cometary CN is ∼90, in line with the Earth and other Solar System objects (including Stardust samples and IDPs), suggesting a common carbon reservoir for the formation of organic matter in the Solar System. However, the ${}^{14}$N/${}^{15}$N ratio in comets is lower than the Earth by a factor of two and lower than the protosolar value by a factor of three [35]. The reason for this variability in the Solar System is not fully understood, but could be related to different nitrogen reservoirs with distinct isotopic ratios due to the isotope-selective photodissociation of N${}_{2}$ in the protosolar disk [131,132,133].

#### 3.9.2. C${}_{2}$

C${}_{2}$ emission is another very bright feature in the optical spectra of comets, with several bands of the Swan system dominating emission at optical wavelengths. The presence of C${}_{2}$ in comets has been known for as long as CN, yet its true source still remains a mystery. The leading simple organic parent molecule is C${}_{2}$H${}_{2}$, yet in most comets, C${}_{2}$H${}_{2}$ is less abundant than C${}_{2}$ and cannot account for all the observed C${}_{2}$ (e.g., [23]). While C${}_{2}$H${}_{6}$ is often more abundant than C${}_{2}$, the time scale for photodissociation of C${}_{2}$H${}_{6}$ to result in C${}_{2}$ release is much too slow to be a significant source of C${}_{2}$ [9,33]. Therefore, like CN, CHON particles/carbon-rich dust grains are often cited as the source of C${}_{2}$ [22,23,75]. If accurate, this would make C${}_{2}$ emission a bright, easily accessible proxy for tracing complex hydrocarbons/organic matter in comets. However, the current knowledge of the origin of C${}_{2}$ and how this relates to the abundances of C${}_{2}$H${}_{2}$, C${}_{2}$H${}_{6}$, and other hydrocarbons in comets make the analysis of this nature uncertain.

Studies of C${}_{2}$ in comets and comparison to CN have revealed that about 1/3 of comets are depleted in C${}_{2}$ compared to CN [29,30]. These comets are termed “carbon-chain depleted”, as C${}_{2}$ is expected to trace carbon-chain species. About 2/3 of the depleted comets are JFCs. This may be connected to the relative depletions of the hydrocarbons C${}_{2}$H${}_{2}$ and C${}_{2}$H${}_{6}$ observed in the IR (see Section 3.4 and Section 3.6), but there is no evidence that a comet depleted in C${}_{2}$ is always depleted in C${}_{2}$H${}_{2}$ and/or C${}_{2}$H${}_{6}$ as well. The meaning of the optical taxonomy of comets based on C${}_{2}$ requires a more complete knowledge of the origin of C${}_{2}$ in cometary coma.

#### 3.9.3. C${}_{3}$

Emission from C${}_{3}$ was identified along with C${}_{2}$ and CN in the earliest optical spectra of comets [27], though its true nature eluded identification until the mid-20th century [135]. For this reason, its main emission band at 405 nm is called “the Comet Band”. Very little is known about the nature of C${}_{3}$ release in cometary comae. The best molecular candidate is C${}_{3}$H${}_{4}$ (propyne), though like the case of C${}_{2}$H${}_{6}$ and C${}_{2}$, this is not likely to be a dominant parent [33]. As with C${}_{2}$, CHON particles/carbon-rich dust grains are a possibility. However, it seems that there is not a simple origin that explains the presence of C${}_{2}$ and C${}_{3}$ in cometary comae, as the abundances of C${}_{3}$ and C${}_{2}$ do not always correlate (i.e., some comets are depleted in C${}_{2}$ but have normal C${}_{3}$ abundances and vice versa) [29,30]. Much more work is needed to understand the origin of C${}_{3}$ in cometary comae.

#### 3.9.4. CH

Emissions from CH are harder to detect than CN, C${}_{2}$, and C${}_{3}$ due to the main band observable at optical wavelengths being intrinsically weaker. Therefore, the literature of CH observations in comets is less extensive than these other species, though its presence has been known in comets for nearly as long [136]. The current state of knowledge of CH parentage is similar to C${}_{3}$: CH${}_{4}$ is suggested as a possible parent molecule, though CH release from CH${}_{4}$ photodissociation is a very inefficient process. Like the other radical species discussed in this section, CH release from CHON particles/carbon-rich dust grains is a possibility, but has not been studied in any detail.

## 4. Implications

Remote sensing observations at a variety of wavelengths have revealed a number of organic molecules present in comets. The presence of these relatively simple organic molecules hinted at the potential presence of more complicated species, a supposition confirmed by the Rosetta mission, which greatly expanded the list of known organic molecules in comets. Apart from providing an inventory of organic molecules, these results also have implications for the origin and formation of organic matter, as well as their potential delivery to the terrestrial planets.

**Figure 11:**
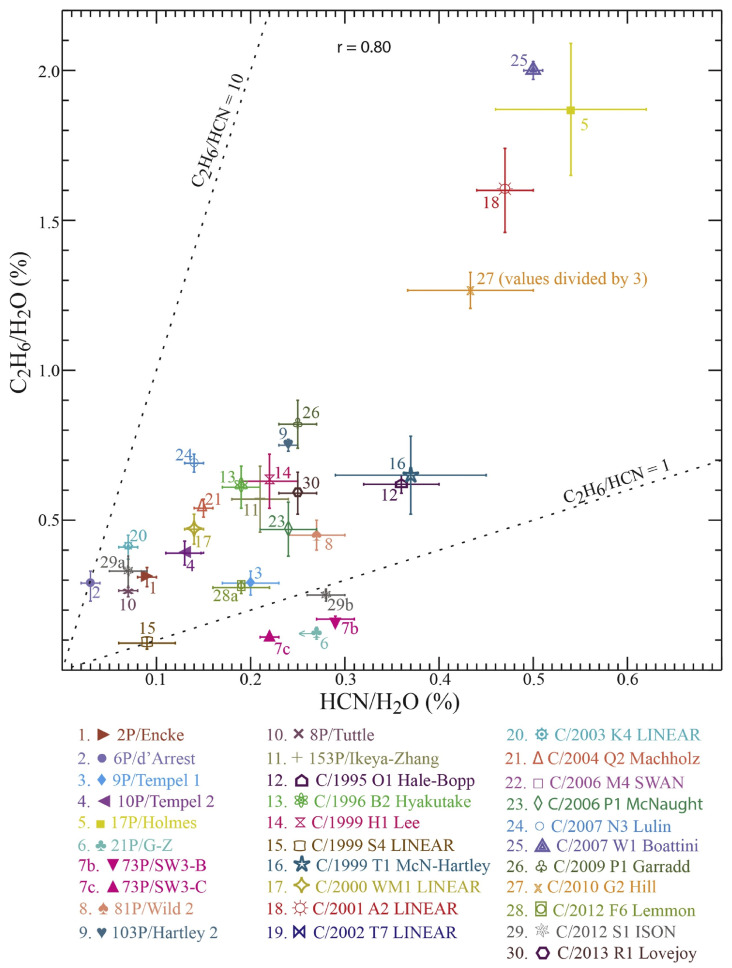
Abundances of C${}_{2}$H${}_{6}$ plotted versus HCN abundances in the sample of comets measured at IR wavelengths. The abundances show a fairly strong positive correlation, suggestive of a common release mechanism. Figure from [8].

Many of the simple organic molecules, such as C${}_{2}$H${}_{6}$, H${}_{2}$CO, and CH${}_{3}$OH, are efficiently formed via hydrogen addition reactions on grain surfaces involving C${}_{2}$H${}_{2}$ and CO. The relative abundances of these species in comets show evidence for this formation mechanism in the protosolar disk. While CO/H${}_{2}$CO/CH${}_{3}$OH ratios show a large scatter suggesting variable efficiency in hydrogenation of CO (or a large role of evolutionary/sublimation effects on observed abundances), C${}_{2}$H${}_{6}$/C${}_{2}$H${}_{2}$ ratios are more constant and suggest a very efficient hydrogenation process for C${}_{2}$H${}_{2}$ on grain surfaces, if this is indeed the formation process for C${}_{2}$H${}_{6}$ in the protosolar disk [8]. Correlations between the abundances of different organic molecules and mapping of spatial distributions in the coma can reveal links between the formation of different organic molecules and their colocation in the nucleus. HCN is highly correlated with hydrocarbons, specifically C${}_{2}$H${}_{6}$, and these species often have similar spatial distributions when observed in the IR, suggesting a common release mechanism from the nucleus (see Figure 11) [8]. Species like H${}_{2}$CO and CH${}_{3}$OH show weaker correlations and different spatial distributions, often indicative of an extended source of production ([8,48], see also Figure 4 and Figure 7). These correlations (or lack thereof) could therefore indicate different ice phases in the nucleus (for instance apolar vs. polar, see Section 3.4), and how the organic molecules segregate into these different ice phases could have ramifications for their formation and incorporation into cometary nuclei.

Several species (in addition to the known photochemical products observed at optical wavelengths discussed in Section 3.9) have distributed sources in cometary comae, including H${}_{2}$CO, HNC, and sometimes CH${}_{3}$OH. While for CH${}_{3}$OH this may point to incorporation of this molecule into water-rich ice grains [96,97,99], the distributed sources for H${}_{2}$CO and HNC point to a more complex progenitor contained in the cometary dust ([48,49] Section 3.1 and Section 3.2). Understanding the nature of these distributed sources can yield clues to the nature of complex organic material in cometary dust grains.

The organic content of comets suggests they could be an important source of the Earth’s current biosphere. Using the measurements of organic matter in comet 67P by Rosetta, Rubin et al. 2019 [137] and Altwegg et al. 2019 [65] argued that comets could account for all of the organic material currently present in Earth’s biosphere.

The presence of complex organic matter in comets suggests that these molecules form readily in protoplanetary disks and/or the ISM. The organic inventory of comets exceeds the complexity of the ISM, which could point to interstellar inheritance as an origin for some Solar System ices. In particular, the presence of glycine in both Rosetta observations and the Stardust samples suggests amino acids can form in a protoplanetary disk/ISM environment.

## 5. Future Directions for the Study of Cometary Organic Matter

Both remote sensing observations and directed missions to comets have provided unique insights into cometary organic matter, and both types of studies continue to be vital to provide further understanding. Remote sensing observations are limited to simpler organic molecules, but provide the statistical sampling needed to understand the cometary population as a whole. On the other hand, space missions provide detailed analysis of a specific target, quantifying organic matter that can currently only be detected by being in close proximity (e.g., glycine).

Future missions to comets will build upon the success of Rosetta and Stardust. Comet Interceptor is an ESA mission that will perform a flyby of either a dynamically new (first passage through the inner Solar System) or interstellar object to be determined, providing the first close up study of any comet in either of these dynamical classes, though as a flyby it will be more limited in the scope of its investigations than Rosetta [138]. The next major step in the study of cometary organic molecules is to build off the success of Stardust and Philae and return a sample of material directly from the cometary surface, preferably through a cryogenic sample return, in order to preserve the structure of the nucleus ices. The Comet Astrobiology Exploration Sample Return (CAESAR) was a proposed mission to the Rosetta target comet 67P in order to obtain a sample from the surface and bring it back to Earth. This mission was a finalist in the latest call for NASA New Frontiers Class proposals but was not selected. A similar mission concept called AMBITION has been proposed as part of ESA’s Voyage 2050 program [139].There is no currently selected cometary sample return mission planned for launch, cryogenic or otherwise. In the meantime, analysis of the vast amount of data obtained by Rosetta will continue, providing new insights into the composition of cometary organic matter.

The next generation of remote sensing facilities will provide similar leaps forward in our understanding of cometary organic matter. ALMA has already provided detailed studies of species such as H${}_{2}$CO, CH${}_{3}$OH, and HCN, and will continue to do so. Sub-mm observations to search for the more complicated organic molecules detected by ROSINA will be able to identify different isomers (for instance dimethyl ether (CH${}_{3}$OCH${}_{3}$) and ethanol), which could not be differentiated with ROSINA. The iSHELL instrument on the NASA IRTF has provided similarly vast improvements in the ability to quantify the composition of comets at IR wavelengths. The next generation of 30-m class telescopes will extend remote sensing observations of cometary volatiles to much fainter targets than currently possible. The James Webb Space Telescope (JWST) will be able to observe the aromatic and aliphatic C–H stretch transitions from hydrocarbons in the 3.3–3.4 micron region [140]. Recent observations of the interstellar comet 2I/Borisov gave us our first insight into the organic composition of comets from other star systems. While some deviations from Solar System comets have been noted (C${}_{2}$ depleted, CO enriched), 2I/Borisov is overall fairly similar [141,142,143,144]. With survey telescopes such as the Vera Rubin Observatory going online in coming years, more opportunities to discover and characterize the organic composition of interstellar comets should follow with more new discoveries.

Specific avenues for further study include (but are not limited to):

(1) Release of organic matter into the coma.Past results at IR and sub-mm wavelengths have shown that while many organic molecules are released directly from the nucleus (C${}_{2}$H${}_{6}$, HCN), others have a distributed source. H${}_{2}$CO has a wide range of abundances in comets, and ALMA observations have shown distributed sources for multiple comets to date. Further observations are needed to understand H${}_{2}$CO abundances in comets and how they connect to more complex organic matter such as POM, CHON particles, or polymers. Similarly, the origin of HNC is not well understood, though it is clear that it is not released directly from the nucleus. The identity of the CH${}_{3}$OH extended source observed for some comets also warrants further study. A detailed study of the scattering properties of dust grains can also reveal the presence of organic matter [145,146].

(2) Further development of compositional taxonomies. While the optical taxonomy [29,30] has a large sample size (>200 comets), its meaning for the organic composition of comets is not well understood due to a lack of knowledge of the origin of the observed radicals. On the other hand, IR and sub-mm abundance patterns are more straightforward to interpret, but suffer from small sample sizes (∼50). While trends are emerging [8], the recent discovery of C/2016 R2 (PanSTARRS), a comet dominated by CO with high N${}_{2}$ and low H${}_{2}$O abundances [147,148,149,150,151], has revealed that there is still much that is not understood about cometary composition. More observations at all wavelengths are needed to better interpret compositional taxonomies and what they imply about the organic composition of cometary nuclei.

(3) Cryogenic sample return.The next giant leap in the study of cometary organic matter will be the return of a (preferably cryogenic) sample to laboratories on Earth through a mission such as AMBITION, as discussed above. This will enable the detection and characterization of complex organic matter that has been measured in meteorites, but will only be studied in comets through a sample that is returned to Earth.

Comets are rich in organic matter and are vital tools to understanding the formation of organic matter in the ISM/protosolar disk and subsequent delivery to the terrestrial planets. Past studies have revealed much about the organic inventory of comets, and the future is bright for continued insights into the organic composition of cometary nuclei.

## Figures and Tables

**Table 1:** Organic molecules detected in comets via Earth-based remote sensing. Does not include species only detected by Rosetta at 67P, though Rosetta measurements are included in the statistics. Based on compilation of Dello Russo et al. 2016 [8] and Bockelee-Morvan and Biver 2017 [67], updated with more recent observations as of July 2020. See Table A1 for the list of comets with recent observations.

Species	Chemical Formula	Typical Abundance (X/H${}_{2}$O in %)	Number of Comets
Hydrogen Cyanide	HCN	0.1–0.2 ^a^	57
Hydrogen Isocyanide	HNC	0.01	21
Isocyanic Acid	HNCO	0.02	16
Acetonitrile	CH${}_{3}$CN	0.02	22
Cyanoacetylene	HC${}_{3}$N	0.02	11
Acetylene	C${}_{2}$H${}_{2}$	0.13	30
Methane	CH${}_{4}$	0.72	32
Ethane	C${}_{2}$H${}_{6}$	0.52	35
Formaldehyde	H${}_{2}$CO	0.30	31
Methanol	CH${}_{3}$OH	2.05	35
Formic Acid	HCOOH	0.09	10
Methyl Formate	HCOOCH${}_{3}$	0.003–0.08	3
Acetaldehyde	CH${}_{3}$CHO	0.05–0.08	4
Ethylene Glycol	(CH${}_{2}$OH)${}_{2}$	0.01–0.35	5
Ethanol	C${}_{2}$H${}_{5}$OH	0.04–0.12	2
Glycolaldehyde	CH${}_{2}$OHCHO	0.02	2
Formamide	NH${}_{2}$CHO	0.004–0.021	5
Thioformaldehyde	H${}_{2}$CS	0.003–0.09	4

^a^ The mean value for sub-mm observations is 0.1%, while the mean value for IR observations is 0.2%. See Section 3.1

## Data Availability

Not applicable.

## Appendix A

This appendix includes individual comets observed after 2015 that we included in Table 1 and were not included in the previous compilations of Dello Russo et al. 2016 [8] and Bockelee-Morvan and Biver 2017 [67]. For the statistics for results published prior to 2015, we refer the reader to these works. Any species present in Table 1 but not below do not have any new ground-based observations reported since 2015. Blank columns indicate that no new observation (i.e., since 2015) for that species in that particular comet is available. For comets with a range of values from different works, we used the average of these values when calculating mean values for the population. In addition to the species below, Rosetta quantified the following species that have been detected remotely in other comets: HNCO/H${}_{2}$O = 0.027%, CH${}_{3}$CN/H${}_{2}$O = 0.006%, HC${}_{3}$N/H${}_{2}$O = 0.0004%, HCOOH/H${}_{2}$O = 0.013%, HCOOCH${}_{3}$/H${}_{2}$O = 0.003%, CH${}_{3}$CHO/H${}_{2}$O = 0.047%, (CH${}_{2}$OH)${}_{2}$/H${}_{2}$O = 0.011%, C${}_{2}$H${}_{5}$OH/H${}_{2}$O = 0.039%, NH${}_{2}$CHO/H${}_{2}$O = 0.004%, and H${}_{2}$CS/H${}_{2}$O = 0.0027% [137,152,153].

**Table A1:** Table detailing abundances observed in individual comets since 2015 that were included in the tabulation of Table 1

Comet	Abundance (X/H${}_{2}$O in %)						
	HCN	HNC	C${}_{2}$H${}_{2}$	CH${}_{4}$	C${}_{2}$H${}_{6}$	H${}_{2}$CO	CH${}_{3}$OH
2P/Encke [110]	0.12			0.11	0.04	0.27	0.87
21P/Giacobini-Zinner [111,114]	0.15			1.1	0.23		2.3
45P/Honda-Mrkos-Pajdušáková [52,113]	0.05–0.15		0.08	0.79–1.0	0.52–0.81	0.14–0.36	3.6–4.5
46P/Wirtanen [154]	0.09						
67P/Churymov-Gerasimenko [108,127,137,153]	0.14			0.34–0.47	0.29	0.32	0.21
252P/LINEAR [155]	0.23				0.95		4.9
C/2012 F6 (Lemmon) [156]	0.06	0.003				0.1	0.97
C/2012 K1 (PanSTARRS) [50,157]	0.14			0.46	0.87		1.7–2.7
C/2012 S1 (ISON) [75,156,158]	0.11–0.26	0.05	0.21	0.33	0.29	0.23–1.1	0.49–1.4
C/2013 R1 (Lovejoy) [159]	0.2						
C/2013 V5 (Oukaimeden) [107]	0.08		0.07	0.28	0.2	0.13	0.95
C/2014 Q2 (Lovejoy) [159,160]	0.09					0.2	2.2
C/2017 E4 (Lovejoy) [161]	0.17		0.14	0.34	0.39	0.36	1.62

## References

[1] Altwegg K., Balsiger H., Berthelier J.J., Bieler A., Calmonte U., De Keyser J., Fiethe B., Fuselier S.A., Gasc S., Gombosi T.I. (2017). D_2_O and HDS in the coma of 67P/Churyumov-Gerasimenko. Philos. Trans. R. Soc. Lond. Ser. A.

[2] Eistrup C., Walsh C., van Dishoeck E.F. (2019). Cometary compositions compared with protoplanetary disk midplane chemical evolution. An emerging chemical evolution taxonomy for comets. Astron. Astrophys..

[3] Whipple F.L. (1950). A comet model. I. The acceleration of Comet Encke. Astorphys. J..

[4] Fulle M., Blum J., Green S.F., Gundlach B., Herique A., Moreno F., Mottola S., Rotundi A., Snodgrass C. (2019). The refractory-to-ice mass ratio in comets. Mon. Not. R. Astron. Soc..

[5] Mumma M.J., Charnley S.B. (2011). The Chemical Composition of Comets—Emerging Taxonomies and Natal Heritage. Annu. Rev. Astonomy Astrophys..

[6] Cochran A.L., Levasseur-Regourd A.C., Cordiner M., Hadamcik E., Lasue J., Gicquel A., Schleicher D.G., Charnley S.B., Mumma M.J., Paganini L. (2015). The Composition of Comets. Space Sci. Rev..

[7] Bieler A., Altwegg K., Balsiger H., Bar-Nun A., Berthelier J.J., Bochsler P., Briois C., Calmonte U., Combi M., de Keyser J. (2015). Abundant molecular oxygen in the coma of comet 67P/Churyumov-Gerasimenko. Nature.

[8] Dello Russo N., Kawakita H., Vervack R.J., Weaver H.A. (2016). Emerging trends and a comet taxonomy based on the volatile chemistry measured in thirty comets with high-resolution infrared spectroscopy between 1997 and 2013. Icarus.

[9] Helbert J., Rauer H., Boice D.C., Huebner W.F. (2005). The chemistry of C_2_ and C_3_ in the coma of Comet C/1995 O1 (Hale-Bopp) at heliocentric distances r_h_≥ 2.9 AU. Astron. Astrophys..

[10] Biver N., Bockelée-Morvan D., Moreno R., Crovisier J., Colom P., Lis D.C., Sandqvist A., Boissier J., Despois D., Milam S.N. (2015). Ethyl alcohol and sugar in comet C/2014 Q2 (Lovejoy). Sci. Adv..

[11] Rivilla V.M., Drozdovskaya M.N., Altwegg K., Caselli P., Beltrán M.T., Fontani F., van der Tak F.F.S., Cesaroni R., Vasyunin A., Rubin M. (2020). ALMA and ROSINA detections of phosphorus-bearing molecules: The interstellar thread between star-forming regions and comets. Mon. Not. R. Astron. Soc..

[12] Altwegg K., Balsiger H., Bar-Nun A., Berthelier J.J., Bieler A., Bochsler P., Briois C., Calmonte U., Combi M.R., Cottin H. (2016). Prebiotic chemicals–amino acid and phosphorus–in the coma of comet 67P/Churyumov-Gerasimenko. Sci. Adv..

[13] Combes M., Crovisier J., Encrenaz T., Moroz V.I., Bibring J.P. (1988). The 2.5–12 micron spectrum of Comet Halley from the IKS-VEGA Experiment. Icarus.

[14] Moreels G., Clairemidi J., Hermine P., Brechignac P., Rousselot P. (1994). Detection of a polycyclic aromatic molecule in comet P/Halley. Astron. Astrophys..

[15] Lisse C.M., VanCleve J., Adams A.C., A’Hearn M.F., Fernández Y.R., Farnham T.L., Armus L., Grillmair C.J., Ingalls J., Belton M.J.S. (2006). Spitzer Spectral Observations of the Deep Impact Ejecta. Science.

[16] Sandford S.A., Aléon J., Alexand er C.M.O.D., Araki T., Bajt S., Baratta G.A., Borg J., Bradley J.P., Brownlee D.E., Brucato J.R. (2006). Organics Captured from Comet 81P/Wild 2 by the Stardust Spacecraft. Science.

[17] Clairemidi J., Bréchignac P., Moreels G., Pautet D. (2004). Tentative identification of pyrene as a polycyclic aromatic molecule in UV spectra of comet P/Halley: An emission from 368 to 384 nm. Planet. Space Sci..

[18] Clairemidi J., Moreels G., Mousis O., Bréchignac P. (2008). Identification of anthracene in Comet 1P/Halley. Astron. Astrophys..

[19] Ootsubo T., Kawakita H., Shinnaka Y., Watanabe J.I., Honda M. (2020). Unidentified infrared emission features in mid-infrared spectrum of comet 21P/Giacobini-Zinner. Icarus.

[20] Altwegg K., Balsiger H., Berthelier J.J., Bieler A., Calmonte U., Fuselier S.A., Goesmann F., Gasc S., Gombosi T.I., Le Roy L. (2017). Organics in comet 67P—A first comparative analysis of mass spectra from ROSINA-DFMS, COSAC and Ptolemy. Mon. Not. R. Astron. Soc..

[21] Clark B.C., Mason L.W., Kissel J. (1987). Systematics of the CHON and Other Light Element Particle Populations in Comet p/Halley. Astron. Astrophys..

[22] Combi M.R., Fink U. (1997). A Critical Study of Molecular Photodissociation and CHON Grain Sources for Cometary C 2. Astrophys. J..

[23] McKay A.J., Chanover N.J., DiSanti M.A., Morgenthaler J.P., Cochran A.L., Harris W.M., Russo N.D. (2014). Rotational variation of daughter species production rates in Comet 103P/Hartley: Implications for the progeny of daughter species and the degree of chemical heterogeneity. Icarus.

[24] Milam S.N., Remijan A.J., Womack M., Abrell L., Ziurys L.M., Wyckoff S., Apponi A.J., Friedel D.N., Snyder L.E., Veal J.M. (2006). Formaldehyde in Comets C/1995 O1 (Hale-Bopp), C/2002 T7 (LINEAR), and C/2001 Q4 (NEAT): Investigating the Cometary Origin of H_2_CO. Astorphysical J..

[25] Donati G.B. (1864). Schreiben des Herrn Prof. Donati an den Herausgeber. Astron. Nachrichten.

[26] Huggins W. (1868). Further Observations on the Spectra of Some of the Stars and Nebulae, with an Attempt to Determine Therefrom Whether These Bodies are Moving towards or from the Earth, Also Observations on the Spectra of the Sun and of Comet II., 1868. Philos. Trans. R. Soc. Lond. Ser. I.

[27] Draper H. (1881). Note on photographs of the spectrum of comet b 1881. Obs.

[28] Farnham T.L., Schleicher D.G., A’Hearn M.F. (2000). The HB Narrowband Comet Filters: Standard Stars and Calibrations. Icarus.

[29] Cochran A.L., Barker E.S., Gray C.L. (2012). Thirty years of cometary spectroscopy from McDonald Observatory. Icarus.

[30] A’Hearn M.F., Millis R.L., Schleicher D.G., Osip D.J., Birch P.V. (1995). The ensemble properties of comets: Results from narrowband photometry of 85 comets, 1976–1992. Icarus.

[31] Fink U. (2009). A taxonomic survey of comet composition 1985–2004 using CCD spectroscopy. Icarus.

[32] Schleicher D., Bair A., Muinonen K., Penttilä A., Granvik M., Virkki A., Fedorets G., Wilkman O., Kohout T. (2014). Chemical and physical properties of comets in the Lowell database: Results from 35 years of narrow-band photometry. Asteroids, Comets, Meteors 2014.

[33] Weiler M. (2012). The chemistry of C_3_ and C_2_ in cometary comae. I. Current models revisited. Astron. Astrophys..

[34] Wyckoff S., Lindholm E., Wehinger P.A., Peterson B.A., Zucconi J.M., Festou M.C. (1989). The C-12/C-13 abundance ratio in Comet Halley. Astrophys. J..

[35] Manfroid J., Jehin E., Hutsemékers D., Cochran A., Zucconi J.M., Arpigny C., Schulz R., Stüwe J.A., Ilyin I. (2009). The CN isotopic ratios in comets. Astron. Astrophys..

[36] Wyckoff S., Kleine M., Peterson B.A., Wehinger P.A., Ziurys L.M. (2000). Carbon Isotope Abundances in Comets. Astrophys. J..

[37] Rousselot P., Pirali O., Jehin E., Vervloet M., Hutsemékers D., Manfroid J., Cordier D., Martin-Drumel M.A., Gruet S., Arpigny C. (2014). Toward a Unique Nitrogen Isotopic Ratio in Cometary Ices. Astrophys. J. Lett..

[38] Shinnaka Y., Kawakita H., Kobayashi H., Nagashima M., Boice D.C. (2014). ^14^NH_2_/^15^NH_2_ Ratio in Comet C/2012 S1 (ISON) Observed during its Outburst in 2013 November. Astrophys. J. Lett..

[39] Shinnaka Y., Kawakita H., Jehin E., Decock A., Hutsemékers D., Manfroid J., Arai A. (2016). Nitrogen isotopic ratios of NH_2_ in comets: Implication for ^15^N-fractionation in cometary ammonia. Mon. Not. R. Astron. Soc..

[40] Mumma M.J., Weaver H.A., Larson H.P., Williams M., Davis D.S. (1986). Detection of water vapor in Halley’s comet. Science.

[41] Dello Russo N., DiSanti M.A., Mumma M.J., Magee-Sauer K., Rettig T.W. (1998). Carbonyl Sulfide in Comets C/1996 B2 (Hyakutake) and C/1995 O1 (Hale-Bopp): Evidence for an Extended Source in Hale-Bopp. Icarus.

[42] Mumma M.J., Dello Russo N., DiSanti M.A., Magee-Sauer K., Novak R.E., Brittain S., Rettig T., McLean I.S., Reuter D.C., Xu L.H. (2001). Organic Composition of C/1999 S4 (LINEAR): A Comet Formed Near Jupiter?. Science.

[43] Turner B.E. (1974). Detection of OH at 18-CENTIMETER Wavelength in Comet Kohoutek (1973f). Astrophys. J..

[44] Huebner W.F., Snyder L.E., Buhl D. (1974). HCN Radio Emission from Comet Kohoutek (1973f). Icarus.

[45] Biver N., Bockelée-Morvan D., Crovisier J., Davies J.K., Matthews H.E., Wink J.E., Rauer H., Colom P., Dent W.R.F., Despois D. (1999). Spectroscopic Monitoring of Comet C/1996 B2 (Hyakutake) with the JCMT and IRAM Radio Telescopes. Astron. J..

[46] Biver N., Bockelée-Morvan D., Colom P., Crovisier J., Henry F., Lellouch E., Winnberg A., Johansson L., Gunnarsson M., Rickman H. (2002). The 1995–2002 Long-Term Monitoring of Comet C/1995 O1 (Hale-Bopp) at Radio Wavelength. Earth Moon Planets.

[47] Biver N., Bockelée-Morvan D., Crovisier J., Lis D.C., Moreno R., Colom P., Henry F., Herpin F., Paubert G., Womack M. (2006). Radio wavelength molecular observations of comets C/1999 T1 (McNaught-Hartley), C/2001 A2 (LINEAR), C/2000 WM_1_ (LINEAR) and 153P/Ikeya-Zhang. Astron. Astrophys..

[48] Cordiner M.A., Remijan A.J., Boissier J., Milam S.N., Mumma M.J., Charnley S.B., Paganini L., Villanueva G., Bockelée-Morvan D., Kuan Y.J. (2014). Mapping the Release of Volatiles in the Inner Comae of Comets C/2012 F6 (Lemmon) and C/2012 S1 (ISON) Using the Atacama Large Millimeter/Submillimeter Array. Astrophys. J..

[49] Cordiner M.A., Boissier J., Charnley S.B., Remijan A.J., Mumma M.J., Villanueva G.L., Lis D.C., Milam S.N., Paganini L., Crovisier J. (2017). ALMA Mapping of Rapid Gas and Dust Variations in Comet C/2012 S1 (ISON):New Insights into the Origin of Cometary HNC. Astrophys. J..

[50] Cordiner M.A., Biver N., Crovisier J., Bockelée-Morvan D., Mumma M.J., Charnley S.B., Villanueva G.L., Paganini L., Lis D.C., Milam S.N. (2017). Thermal Physics of the Inner Coma: ALMA Studies of the Methanol Distribution and Excitation in Comet C/2012 K1 (PanSTARRS). Astrophys. J..

[51] Krankowsky D., Lammerzahl P., Herrwerth I., Woweries J., Eberhardt P., Dolder U., Herrmann U., Schulte W., Berthelier J.J., Illiano J.M. (1986). In situ gas and ion measurements at comet Halley. Nature.

[52] DiSanti M.A., Bonev B.P., Dello Russo N., Vervack R.J., Gibb E.L., Roth N.X., McKay A.J., Kawakita H., Feaga L.M., Weaver H.A. (2017). Hypervolatiles in a Jupiter-family Comet: Observations of 45P/Honda-Mrkos-Pajdušáková Using iSHELL at the NASA-IRTF. Astron. J..

[53] Flynn G.J., Bleuet P., Borg J., Bradley J.P., Brenker F.E., Brennan S., Bridges J., Brownlee D.E., Bullock E.S., Burghammer M. (2006). Elemental Compositions of Comet 81P/Wild 2 Samples Collected by Stardust. Science.

[54] Ogliore R.C., Nagashima K., Huss G.R., Westphal A.J., Gainsforth Z., Butterworth A.L. (2015). Oxygen isotopic composition of coarse- and fine-grained material from comet 81P/Wild 2. Geochim. Cosmochim. Acta.

[55] Elsila J.E., Glavin D.P., Dworkin J.P. (2009). Cometary glycine detected in samples returned by Stardust. Meteorit. Planet. Sci..

[56] Flynn G.J., Keller L.P., Feser M., Wirick S., Jacobsen C. (2003). The origin of organic matter in the solar system: Evidence from the interplanetary dust particles. Geochim. Cosmochim. Acta.

[57] Starkey N.A., Franchi I.A., Lee M.R. (2014). Isotopic diversity in interplanetary dust particles and preservation of extreme ^16^O-depletion. Geochim. Cosmochim. Acta.

[58] Busemann H., Nguyen A.N., Cody G.D., Hoppe P., Kilcoyne A.L.D., Stroud R.M., Zega T.J., Nittler L.R. (2009). Ultra-primitive interplanetary dust particles from the comet 26P/Grigg-Skjellerup dust stream collection. Earth Planet. Sci. Lett..

[59] Boehnhardt H., Bibring J.P., Apathy I., Auster H.U., Ercoli Finzi A., Goesmann F., Klingelhöfer G., Knapmeyer M., Kofman W., Krüger H. (2017). The Philae lander mission and science overview. Philos. Trans. R. Soc. Lond. Ser. A.

[60] De Sanctis M.C., Capaccioni F., Ciarniello M., Filacchione G., Formisano M., Mottola S., Raponi A., Tosi F., Bockelée-Morvan D., Erard S. (2015). The diurnal cycle of water ice on comet 67P/Churyumov-Gerasimenko. Nature.

[61] Filacchione G., de Sanctis M.C., Capaccioni F., Raponi A., Tosi F., Ciarniello M., Cerroni P., Piccioni G., Capria M.T., Palomba E. (2016). Exposed water ice on the nucleus of comet 67P/Churyumov-Gerasimenko. Nature.

[62] Filacchione G., Raponi A., Capaccioni F., Ciarniello M., Tosi F., Capria M.T., De Sanctis M.C., Migliorini A., Piccioni G., Cerroni P. (2016). Seasonal exposure of carbon dioxide ice on the nucleus of comet 67P/Churyumov-Gerasimenko. Science.

[63] Quirico E., Moroz L.V., Schmitt B., Arnold G., Faure M., Beck P., Bonal L., Ciarniello M., Capaccioni F., Filacchione G. (2016). Refractory and semi-volatile organics at the surface of comet 67P/Churyumov-Gerasimenko: Insights from the VIRTIS/Rosetta imaging spectrometer. Icarus.

[64] Raponi A., Ciarniello M., Capaccioni F., Mennella V., Filacchione G., Vinogradoff V., Poch O., Beck P., Quirico E., De Sanctis M.C. (2020). Infrared detection of aliphatic organics on a cometary nucleus. Nat. Astron..

[65] Altwegg K., Balsiger H., Fuselier S.A. (2019). Cometary Chemistry and the Origin of Icy Solar System Bodies: The View after Rosetta. Annu. Rev. Astron. Astrophys..

[66] Bockelée-Morvan D., Calmonte U., Charnley S., Duprat J., Engrand C., Gicquel A., Hässig M., Jehin E., Kawakita H., Marty B. (2015). Cometary Isotopic Measurements. Space Sci. Rev..

[67] Bockelée-Morvan D., Biver N. (2017). The composition of cometary ices. Philos. Trans. R. Soc. A.

[68] Filacchione G., Groussin O., Herny C., Kappel D., Mottola S., Oklay N., Pommerol A., Wright I., Yoldi Z., Ciarniello M. (2019). Comet 67P/CG Nucleus Composition and Comparison to Other Comets. Space Sci. Rev..

[69] Oro J., Mills T., Lazcano A. (1991). Comets and the formation of biochemical compounds on the primitive Earth A review. Orig. Life Evol. Biosph..

[70] Despois D., Crovisier J., Bockelée-Morvan D., Gerard E., Schraml J. (1986). Observations of hydrogen cyanide in comet halley. Astron. Astrophys..

[71] Schloerb F.P., Kinzel W.M., Swade D.A., Irvine W.M. (1987). Observations of HCN in comet P/Halley. Astron. Astrophys..

[72] Altwegg K., Balsiger H., Hänni N., Rubin M., Schuhmann M., Schroeder I., Sémon T., Wampfler S., Berthelier J.J., Briois C. (2020). Evidence of ammonium salts in comet 67P as explanation for the nitrogen depletion in cometary comae. Nat. Astron..

[73] Poch O., Istiqomah I., Quirico E., Beck P., Schmitt B., Theulé P., Faure A., Hily-Blant P., Bonal L., Raponi A. (2020). Ammonium salts are a reservoir of nitrogen on a cometary nucleus and possibly on some asteroids. Science.

[74] Dello Russo N., Vervack R.J., Weaver H.A., Kawakita H., Kobayashi H., Biver N., Bockelée-Morvan D., Crovisier J. (2009). The Parent Volatile Composition of 6p/d’Arrest and a Chemical Comparison of Jupiter-Family Comets Measured at Infrared Wavelengths. Astrophys. J..

[75] Dello Russo N., Vervack R.J., Kawakita H., Cochran A., McKay A.J., Harris W.M., Weaver H.A., Lisse C.M., DiSanti M.A., Kobayashi H. (2016). The compositional evolution of C/2012 S1 (ISON) from ground-based high-resolution infrared spectroscopy as part of a worldwide observing campaign. Icarus.

[76] Irvine W.M., Bockelée-Morvan D., Lis D.C., Matthews H.E., Biver N., Crovisier J., Davies J.K., Dent W.R.F., Gautier D., Godfrey P.D. (1996). Spectroscopic evidence for interstellar ices in comet Hyakutake. Nature.

[77] Biver N., Bockelée-Morvan D., Colom P., Crovisier J., Germain B., Lellouch E., Davies J.K., Dent W.R.F., Moreno R., Paubert G. (1997). Long-term Evolution of the Outgassing of Comet Hale-Bopp From Radio Observations. Earth Moon Planets.

[78] Rodgers S.D., Charnley S.B. (1998). HNC and HCN in Comets. Astrophys. J. Lett..

[79] Rodgers S.D., Charnley S.B. (2001). On the origin of HNC in Comet Lee. Mon. Not. R. Astron. Soc..

[80] Lis D.C., Bockelée-Morvan D., Boissier J., Crovisier J., Biver N., Charnley S.B. (2008). Hydrogen Isocyanide in Comet 73P/Schwassmann-Wachmann (Fragment B). Astrophys. J..

[81] Bockelée-Morvan D., Colom P., Crovisier J., Despois D., Paubert G. (1991). Microwave detection of hydrogen sulphide and methanol in comet Austin (1989c1). Nature.

[82] Colom P., Crovisier J., Bockelée-Morvan D., Despois D., Paubert G. (1992). Formaldehyde in comets. I - Microwave observations of P/Brorsen-Metcalf (1989 X), Austin (1990 V) and Levy (1990 XX). Astron. Astrophys..

[83] Moroz V.I., Combes M., Bibring J.P., Coron N., Crovisier J., Encrenaz T., Crifo J.F., Sanko N., Grigoryev A.V., Bockelée-Morvan D. (1987). Detection of Parent Molecules in Comet p/ Halley from the IKS VEGA Experiment. Astron. Astrophys..

[84] Danks A.C., Encrenaz T., Bouchet P., Le Bertre T., Chalabaev A. (1987). The spectrum of comet P/Halley from 3.0 to 4.0 microns. Astron. Astrophys..

[85] Snyder L.E., Palmer P., de Pater I. (1989). Radio Detection of Formaldehyde Emission from Comet Halley. Astron. J..

[86] Meier R., Eberhardt P., Krankowsky D., Hodges R.R. (1993). The extended formaldehyde source in comet P/Halley. Astron. Astrophys..

[87] Bockelée-Morvan D., Lis D.C., Wink J.E., Despois D., Crovisier J., Bachiller R., Benford D.J., Biver N., Colom P., Davies J.K. (2000). New molecules found in comet C/1995 O1 (Hale-Bopp). Investigating the link between cometary and interstellar material. Astron. Astrophys..

[88] DiSanti M.A., Bonev B.P., Magee-Sauer K., Dello Russo N., Mumma M.J., Reuter D.C., Villanueva G.L. (2006). Detection of Formaldehyde Emission in Comet C/2002 T7 (LINEAR) at Infrared Wavelengths: Line-by-Line Validation of Modeled Fluorescent Intensities. Astrophys. J..

[89] Cottin H., Fray N. (2008). Distributed Sources in Comets. Space Sci. Rev..

[90] Capaccioni F., Coradini A., Filacchione G., Erard S., Arnold G., Drossart P., De Sanctis M.C., Bockelée-Morvan D., Capria M.T., Tosi F. (2015). The organic-rich surface of comet 67P/Churyumov-Gerasimenko as seen by VIRTIS/Rosetta. Science.

[91] Fray N., Bénilan Y., Biver N., Bockelée-Morvan D., Cottin H., Crovisier J., Gazeau M.C. (2006). Heliocentric evolution of the degradation of polyoxymethylene: Application to the origin of the formaldehyde (H 2CO) extended source in Comet C/1995 O1 (Hale-Bopp). Icarus.

[92] Garrod R.T., Widicus Weaver S.L., Herbst E. (2008). Complex Chemistry in Star-forming Regions: An Expanded Gas-Grain Warm-up Chemical Model. Astrophys. J..

[93] Öberg K.I., Garrod R.T., van Dishoeck E.F., Linnartz H. (2009). Formation rates of complex organics in UV irradiated CH_3_OH-rich ices. I. Experiments. Astron. Astrophys..

[94] Herbst E., van Dishoeck E.F. (2009). Complex Organic Interstellar Molecules. Annu. Rev. Astron. Astrophys..

[95] Bockelée-Morvan D., Crovisier J., Colom P., Despois D. (1994). The rotational lines of methanol in comets Austin 1990 V and Levy 1990 XX. Astron. Astrophys..

[96] Dello Russo N., Vervack R.J., Lisse C.M., Weaver H.A., Kawakita H., Kobayashi H., Cochran A.L., Harris W.M., McKay A.J., Biver N. (2011). The Volatile Composition and Activity of Comet 103P/Hartley 2 During the EPOXI Closest Approach. Astrophys. J. Lett..

[97] Mumma M.J., Bonev B.P., Villanueva G.L., Paganini L., DiSanti M.A., Gibb E.L., Keane J.V., Meech K.J., Blake G.A., Ellis R.S. (2011). Temporal and Spatial Aspects of Gas Release During the 2010 Apparition of Comet 103P/Hartley 2. Astrophys. J..

[98] Bonev B.P., Villanueva G.L., Paganini L., DiSanti M.A., Gibb E.L., Keane J.V., Meech K.J., Mumma M.J. (2013). Evidence for two modes of water release in Comet 103P/Hartley 2: Distributions of column density, rotational temperature, and ortho-para ratio. Icarus.

[99] Kawakita H., Kobayashi H., Dello Russo N., Vervack R.J., Hashimoto M., Weaver H.A., Lisse C.M., Cochran A.L., Harris W.M., Bockelée-Morvan D. (2013). Parent volatiles in Comet 103P/Hartley 2 observed by Keck II with NIRSPEC during the 2010 apparition. Icarus.

[100] Villanueva G.L., Mumma M.J., DiSanti M.A., Bonev B.P., Gibb E.L., Magee-Sauer K., Blake G.A., Salyk C. (2011). The molecular composition of Comet C/2007 W1 (Boattini): Evidence of a peculiar outgassing and a rich chemistry. Icarus.

[101] Mumma M.J., DiSanti M.A., dello Russo N., Fomenkova M., Magee-Sauer K., Kaminski C.D., Xie D.X. (1996). Detection of Abundant Ethane and Methane, Along with Carbon Monoxide and Water, in Comet C/1996 B2 Hyakutake: Evidence for Interstellar Origin. Science.

[102] Tielens A.G.G.M., Bohme D.K. (1992). Grain Surface Chemistry. Chemistry and Spectroscopy of Interstellar Molecules.

[103] Kobayashi H., Hidaka H., Lamberts T., Hama T., Kawakita H., Kästner J., Watanabe N. (2017). Hydrogenation and Deuteration of C_2_H_2_ and C_2_H_4_ on Cold Grains: A Clue to the Formation Mechanism of C_2_H_6_ with Astronomical Interest. Astrophys. J..

[104] Gerakines P.A., Schutte W.A., Ehrenfreund P. (1996). Ultraviolet processing of interstellar ice analogs. I. Pure ices. Astron. Astrophys..

[105] Bennett C.J., Jamieson C.S., Osamura Y., Kaiser R.I. (2006). Laboratory Studies on the Irradiation of Methane in Interstellar, Cometary, and Solar System Ices. Astrophys. J..

[106] De Barros A.L.F., da Silveira E.F., Fulvio D., Rothard H., Boduch P. (2016). Ion Irradiation of Ethane and Water Mixture Ice at 15 K: Implications for the Solar System and the ISM. Astrophys. J..

[107] DiSanti M.A., Bonev B.P., Gibb E.L., Roth N.X., Dello Russo N., Vervack R.J. (2018). Comet C/2013 V5 (Oukaimeden): Evidence for Depleted Organic Volatiles and Compositional Heterogeneity as Revealed through Infrared Spectroscopy. Astron. J..

[108] Bockelée-Morvan D., Crovisier J., Erard S., Capaccioni F., Leyrat C., Filacchione G., Drossart P., Encrenaz T., Biver N., de Sanctis M.C. (2016). Evolution of CO_2_, CH_4_, and OCS abundances relative to H_2_O in the coma of comet 67P around perihelion from Rosetta/VIRTIS-H observations. Mon. Not. R. Astron. Soc..

[109] Le Roy L., Altwegg K., Balsiger H., Berthelier J.J., Bieler A., Briois C., Calmonte U., Combi M.R., De Keyser J., Dhooghe F. (2015). Inventory of the volatiles on comet 67P/Churyumov-Gerasimenko from Rosetta/ROSINA. Astron. Astrophys..

[110] Roth N.X., Gibb E.L., Bonev B.P., DiSanti M.A., Dello Russo N., Vervack R.J., McKay A.J., Kawakita H. (2018). A Tale of “Two” Comets: The Primary Volatile Composition of Comet 2P/Encke Across Apparitions and Implications for Cometary Science. Astron. J..

[111] Faggi S., Mumma M.J., Villanueva G.L., Paganini L., Lippi M. (2019). Quantifying the Evolution of Molecular Production Rates of Comet 21P/Giacobini—Zinner with iSHELL/NASA-IRTF. Astron. J..

[112] McKay A., DiSanti M., Bonev B., Dello Russo N., Vervack R.J., Gibb E., Roth N., Saki M., Kawakita H. Hypervolatiles in Jupiter Family Comet 46P/Wirtanen Observed with IRTF iSHELL. Proceedings of the EPSC-DPS Joint Meeting 2019.

[113] Dello Russo N., Kawakita H., Bonev B.P., Vervack R.J., Gibb E.L., Shinnaka Y., Roth N.X., DiSanti M.A., McKay A.J. (2020). Post-perihelion volatile production and release from Jupiter-family comet 45P/Honda-Mrkos-Pajdušáková. Icarus.

[114] Roth N.X., Gibb E.L., Bonev B.P., DiSanti M.A., Dello Russo N., McKay A.J., Vervack R.J., Kawakita H., Saki M., Biver N. (2020). Probing the Evolutionary History of Comets: An Investigation of the Hypervolatiles CO, CH_4_, and C_2_H_6_ in the Jupiter-family Comet 21P/Giacobini—Zinner. Astron. J..

[115] Qasim D., Fedoseev G., Chuang K.J., He J., Ioppolo S., van Dishoeck E.F., Linnartz H. (2020). An experimental study of the surface formation of methane in interstellar molecular clouds. Nat. Astron..

[116] Martínez L., Santoro G., Merino P., Accolla M., Lauwaet K., Sobrado J., Sabbah H., Pelaez R.J., Herrero V.J., Tanarro I. (2020). Prevalence of non-aromatic carbonaceous molecules in the inner regions of circumstellar envelopes. Nat. Astron..

[117] Frenklach M., Feigelson E.D. (1989). Formation of Polycyclic Aromatic Hydrocarbons in Circumstellar Envelopes. Astrophys. J..

[118] Cernicharo J. (2004). The Polymerization of Acetylene, Hydrogen Cyanide, and Carbon Chains in the Neutral Layers of Carbon-rich Proto-planetary Nebulae. Astrophys. J..

[119] Biver N., Bockelée-Morvan D., Debout V., Crovisier J., Boissier J., Lis D.C., Dello Russo N., Moreno R., Colom P., Paubert G. (2014). Complex organic molecules in comets C/2012 F6 (Lemmon) and C/2013 R1 (Lovejoy): Detection of ethylene glycol and formamide. Astron. Astrophys..

[120] Crovisier J., Bockelée-Morvan D., Biver N., Colom P., Despois D., Lis D.C. (2004). Ethylene glycol in comet C/1995 O1 (Hale-Bopp). Astron. Astrophys..

[121] Goesmann F., Rosenbauer H., Bredehöft J.H., Cabane M., Ehrenfreund P., Gautier T., Giri C., Krüger H., Le Roy L., MacDermott A.R.J. (2015). Organic compounds on comet 67P/Churyumov-Gerasimenko revealed by COSAC mass spectrometry. Science.

[122] Hadraoui K., Cottin H., Ivanovski S.L., Zapf P., Altwegg K., Benilan Y., Biver N., Della Corte V., Fray N., Lasue J. (2019). Distributed glycine in comet 67P/Churyumov-Gerasimenko. Astron. Astrophys..

[123] Wright I.P., Sheridan S., Barber S.J., Morgan G.H., Andrews D.J., Morse A.D. (2015). CHO-bearing organic compounds at the surface of 67P/Churyumov-Gerasimenko revealed by Ptolemy. Science.

[124] Fray N., Bardyn A., Cottin H., Altwegg K., Baklouti D., Briois C., Colangeli L., Engrand C., Fischer H., Glasmachers A. (2016). High-molecular-weight organic matter in the particles of comet 67P/Churyumov-Gerasimenko. Nature.

[125] A’Hearn M.F., Belton M.J.S., Delamere W.A., Kissel J., Klaasen K.P., McFadden L.A., Meech K.J., Melosh H.J., Schultz P.H., Sunshine J.M. (2005). Deep Impact: Excavating Comet Tempel 1. Science.

[126] Clemett S.J., Sandford S.A., Nakamura-Messenger K., Hörz F., McKay D.S. (2010). Complex aromatic hydrocarbons in Stardust samples collected from comet 81P/Wild 2. Meteorit. Planet. Sci..

[127] Schuhmann M., Altwegg K., Balsiger H., Berthelier J.J., De Keyser J., Fiethe B., Fuselier S.A., Gasc S., Gombosi T.I., Hänni N. (2019). Aliphatic and aromatic hydrocarbons in comet 67P/Churyumov-Gerasimenko seen by ROSINA. Astron. Astrophys..

[128] Gibb E.L., Mumma M.J., Dello Russo N., DiSanti M.A., Magee-Sauer K. (2003). Methane in Oort cloud comets. Icarus.

[129] Swings P. (1941). Complex structure of cometary bands tentatively ascribed to the contour of the solar spectrum. Lick Obs. Bull..

[130] Vaughan C.M., Pierce D.M., Cochran A.L. (2017). Jet Morphology and Coma Analysis of Comet 103P/Hartley 2. Astron. J..

[131] Heays A.N., Visser R., Gredel R., Ubachs W., Lewis B.R., Gibson S.T., van Dishoeck E.F. (2014). Isotope selective photodissociation of N_2_ by the interstellar radiation field and cosmic rays. Astron. Astrophys..

[132] Visser R., Bruderer S., Cazzoletti P., Facchini S., Heays A.N., van Dishoeck E.F. (2018). Nitrogen isotope fractionation in protoplanetary disks. Astron. Astrophys..

[133] Hily-Blant P., Magalhaes de Souza V., Kastner J., Forveille T. (2019). Multiple nitrogen reservoirs in a protoplanetary disk at the epoch of comet and giant planet formation. Astron. Astrophys..

[134] Yang B., Hutsemékers D., Shinnaka Y., Opitom C., Manfroid J., Jehin E., Meech K.J., Hainaut O.R., Keane J.V., Gillon M. (2018). Isotopic ratios in outbursting comet C/2015 ER61. Astron. Astrophys..

[135] Douglas A.E. (1951). Laboratory Studies of the *λ* 4050 Group of Cometary Spectra. Astrophys. J..

[136] Dufay J. (1940). CH Bands in Comet Spectra. Astrophys. J..

[137] Rubin M., Bekaert D.V., Broadley M.W., Drozdovskaya M.N., Wampfler S.F. (2019). Volatile Species in Comet 67P/Churyumov-Gerasimenko: Investigating the Link from the ISM to the Terrestrial Planets. ACS Earth Space Chem..

[138] Snodgrass C., Jones G.H. (2019). The European Space Agency’s Comet Interceptor lies in wait. Nat. Commun..

[139] Bockelée-Morvan D., Filacchione G., Altwegg K., Bianchi E., Bizzarro M., Blum J., Bonal L., Capaccioni F., Codella C., Choukroun M. (2019). AMBITION—Comet Nucleus Cryogenic Sample Return (White paper for ESA’s Voyage 2050 programme). arXiv.

[140] Kelley M.S.P., Woodward C.E., Bodewits D., Farnham T.L., Gudipati M.S., Harker D.E., Hines D.C., Knight M.M., Kolokolova L., Li A. (2016). Cometary Science with the James Webb Space Telescope. Publ. Astron. Soc. Pac..

[141] Opitom C., Fitzsimmons A., Jehin E., Moulane Y., Hainaut O., Meech K.J., Yang B., Snodgrass C., Micheli M., Keane J.V. (2019). 2I/Borisov: A C_2_ depleted interstellar comet. arXiv.

[142] Kareta T., Andrews J., Noonan J.W., Harris W.M., Smith N., O’Brien P., Sharkey B.N.L., Reddy V., Springmann A., Lejoly C. (2020). Carbon Chain Depletion of 2I/Borisov. Astrophys. J..

[143] McKay A.J., Cochran A.L., Dello Russo N., DiSanti M.A. (2020). Detection of a Water Tracer in Interstellar Comet 2I/Borisov. Astrophys. J..

[144] Cordiner M.A., Milam S.N., Biver N., Bockelée-Morvan D., Roth N.X., Bergin E.A., Jehin E., Remijan A.J., Charnley S.B., Mumma M.J. (2020). Unusually high CO abundance of the first active interstellar comet. Nat. Astron..

[145] Frattin E., Cremonese G., Simioni E., Bertini I., Lazzarin M., Ott T., Drolshagen E., La Forgia F., Sierks H., Barbieri C. (2017). Post-perihelion photometry of dust grains in the coma of 67P Churyumov-Gerasimenko. Mon. Not. R. Astron. Soc..

[146] Filacchione G., Capaccioni F., Ciarniello M., Raponi A., Rinaldi G., De Sanctis M.C., Bockelèe-Morvan D., Erard S., Arnold G., Mennella V. (2020). An orbital water-ice cycle on comet 67P from colour changes. Nature.

[147] Cochran A.L., McKay A.J. (2018). Strong CO^+^ and {{∖rm{N}}}_{2}{+} Emission in Comet C/2016 R2 (Pan-STARRS). Astrophys. J..

[148] Wierzchos K., Womack M. (2018). C/2016 R2 (PANSTARRS): A Comet Rich in CO and Depleted in HCN. Astron. J..

[149] Biver N., Bockelée-Morvan D., Paubert G., Moreno R., Crovisier J., Boissier J., Bertrand E., Boussier H., Kugel F., McKay A. (2018). The extraordinary composition of the blue comet C/2016 R2 (PanSTARRS). Astron. Astrophys..

[150] Opitom C., Hutsemékers D., Jehin E., Rousselot P., Pozuelos F.J., Manfroid J., Moulane Y., Gillon M., Benkhaldoun Z. (2019). High resolution optical spectroscopy of the N_2_-rich comet C/2016 R2 (PanSTARRS). Astron. Astrophys..

[151] McKay A.J., DiSanti M.A., Kelley M.S.P., Knight M.M., Womack M., Wierzchos K., Harrington Pinto O., Bonev B., Villanueva G.L., Dello Russo N. (2019). The Peculiar Volatile Composition of CO-dominated Comet C/2016 R2 (PanSTARRS). Astron. J..

[152] Calmonte U., Altwegg K., Balsiger H., Berthelier J.J., Bieler A., Cessateur G., Dhooghe F., van Dishoeck E.F., Fiethe B., Fuselier S.A. (2016). Sulphur-bearing species in the coma of comet 67P/Churyumov-Gerasimenko. Mon. Not. R. Astron. Soc..

[153] Schuhmann M., Altwegg K., Balsiger H., Berthelier J.J., De Keyser J., Fuselier S.A., Gasc S., Gombosi T.I., Hänni N., Rubin M. (2019). CHO-bearing molecules in Comet 67P/Churyumov-Gerasimenko. ACS Earth Space Chem..

[154] Wang Z., Zhang S.B., Tseng W.L., Sun J.X., Liao Y., Ip W.H., Zheng X.W., Wang N., Lu D.R., Chen L. (2020). Observations of the Hydrogen Cyanide in Comet 46P/Wirtanen at a 3.4 mm Wavelength. Astron. J..

[155] Paganini L., Camarca M.N., Mumma M.J., Faggi S., Lippi M., Villanueva G.L. (2019). Observations of Jupiter Family Comet 252P/LINEAR During a Close Approach to Earth Reveal Large Abundances of Methanol and Ethane. Astron. J..

[156] Bøgelund E.G., Hogerheijde M.R. (2017). Exploring the volatile composition of comets C/2012 F6 (Lemmon) and C/2012 S1 (ISON) with ALMA. Astron. Astrophys..

[157] Roth N.X., Gibb E.L., Bonev B.P., DiSanti M.A., Mumma M.J., Villanueva G.L., Paganini L. (2017). The Composition of Comet C/2012 K1 (PanSTARRS) and the Distribution of Primary Volatile Abundances among Comets. Astron. J..

[158] DiSanti M.A., Bonev B.P., Gibb E.L., Paganini L., Villanueva G.L., Mumma M.J., Keane J.V., Blake G.A., Dello Russo N., Meech K.J. (2016). En Route to Destruction: The Evolution in Composition of Ices in Comet D/2012 S1 (ISON) between 1.2 and 0.34 AU from the Sun as Revealed at Infrared Wavelengths. Astrophys. J..

[159] Wirström E.S., Lerner M.S., Källström P., Levinsson A., Olivefors A., Tegehall E. (2016). HCN observations of comets C/2013 R1 (Lovejoy) and C/2014 Q2 (Lovejoy). Astron. Astrophys..

[160] De Val-Borro M., Milam S.N., Cordiner M.A., Charnley S.B., Villanueva G.L., Kuan Y.J. (2018). CO-activity in comet C/2016 R2 (PANSTARRS). Astron. Telegr..

[161] Faggi S., Villanueva G.L., Mumma M.J., Paganini L. (2018). The Volatile Composition of Comet C/2017 E4 (Lovejoy) before its Disruption, as Revealed by High-resolution Infrared Spectroscopy with iSHELL at the NASA/IRTF. Astron. J..

